# Corynoxine Inhibits Tumor Growth via Targeting NQO1 and Modulating the PTPA–NQO1/PP2A Switch to Activate PP2A

**DOI:** 10.1002/advs.77362

**Published:** 2026-08-20

**Authors:** Guoqing Hou, Li Lin, Mengjie Xia, Ruohan Liang, Yueyue Zhang, Linna Du, Xuan Cao

**Affiliations:** ^1^ School of Medicine Taizhou University Taizhou Zhejiang China; ^2^ The Affiliated Cancer Hospital of Zhengzhou University & Henan Cancer Hospital Zhengzhou Henan China; ^3^ Department of Basic Medicine and Medical Laboratory Science School of Medicine Zhejiang Key Laboratory of New Drug Development for Central Nervous System Diseases Taizhou University Taizhou Zhejiang China

**Keywords:** corynoxine, NQO1, PP2A, PTPA, tumor growth

## Abstract

Corynoxine (Cory) has been reported to suppress lung cancer progression by activating PP2A, but its molecular targets remain unclear. This study shows that Cory exerts anticancer activity across multiple cancer cell lines. Using compound derivatization, affinity‐based protein fishing, LC‐MS/MS, SPR, CETSA, DARTS, and molecular docking, we demonstrate that Cory directly interacts with NQO1, with Gly193 and His194 contributing to ligand recognition. NQO1 is overexpressed and implicated in tumor progression in multiple cancers, although its precise mechanisms remain poorly defined. We identified a previously unrecognized interaction between NQO1 and PTPA that may contribute to PP2A suppression in tumor cells. Cory binding perturbs the NQO1–PTPA interface and weakens their cellular association, thereby facilitating PTPA‐dependent PP2A activation. This activation inhibits the PI3K/AKT and MAPK/ERK pathways and suppresses tumor cell proliferation. Critically, Cory's anticancer effects are abolished in NQO1‐deficient cells, highlighting the essential role of NQO1. Cory did not impair NQO1 enzymatic activity, supporting a non‐enzymatic mechanism. In vivo, Cory suppressed tumor growth in nude mice without obvious systemic toxicity. Collectively, these findings suggest that Cory exerts anticancer effects by modulating the PTPA–NQO1/PP2A switch to activate PP2A, positioning Cory as a promising lead compound for targeted therapy.

## Graphical abstract

Corynoxine exerts potent broad‐spectrum anticancer activity by directly targeting NQO1. This interaction dissociates the oncogenic NQO1‐PTPA complex, releasing PTPA to robustly activate the tumor suppressor PP2A. Consequently, downstream Raf/MEK/ERK and PI3K/AKT signaling pathways are suppressed, downregulating c‐Myc and driving tumor regression.

## 1 Introduction

Cancer remains a major threat to human health, with escalating incidence and mortality rates driving the urgent need for novel therapeutic approaches [1]. In recent years, natural products have emerged as a rich source of anticancer drug discovery, owing to their diverse and novel chemical structures, which serve as valuable lead compounds for drug development [2]. Corynoxine (Cory), an indole alkaloid isolated from the medicinal herb *Uncaria rhynchophylla*, has attracted growing scientific interest. Previous research has demonstrated its potential to ameliorate neurodegenerative disorders. More recently, Cory has gained considerable attention due to its potent anticancer activity [3, 4, 5]. Despite these findings, the potential molecular targets of Cory are yet to be fully elucidated. This study aims to explore the specific mechanisms by which Cory targets critical proteins in tumor cells, thereby providing a comprehensive molecular foundation for its potential as a therapeutic agent.

Protein phosphatase 2A (PP2A) is a major serine/threonine phosphatase that functions as a heterotrimeric holoenzyme. It comprises a structural subunit A (PP2Aa), a regulatory subunit B (PP2Ab), and a catalytic subunit C (PP2Ac). Dysregulation and hyperactivation of signaling pathways controlling cell proliferation, metabolism, and immune responses are pivotal to the malignant transformation of normal cells. PP2A serves as a key negative regulator of these aberrant signaling pathways and is widely recognized as a tumor suppressor [6]. Inhibition of PP2A activity or loss of its subunits has been observed in numerous solid tumors and leukemias. PP2A activators, including Forskolin and FTY720, have demonstrated potent anticancer effects in multiple malignancies, such as chronic myeloid leukemia, multiple myeloma, liver cancer, bladder cancer, and prostate cancer [6, 7, 8]. Our previous research demonstrated that Cory suppresses lung cancer growth both in vitro and in vivo by activating PP2A and inhibiting the AKT‐mTOR/GSK3β signaling axis. Given the therapeutic relevance of PP2A activators, it is therefore critical to elucidate the precise molecular mechanisms through which Cory activates PP2A and exerts its broad‐spectrum antitumor effects. The PI3K/AKT and MAPK/ERK signaling pathways are frequently dysregulated in cancers and contribute significantly to malignant progression [9, 10]. PP2A acts as a negative regulator of both pathways by dephosphorylating key components such as PDK1, AKT, MEK, and ERK [11, 12]. PTPA (Protein phosphatase 2A Activator) serves as a critical upstream regulator of PP2A, enhancing its phosphatase activity through a highly conserved mechanism. PTPA binds directly to the catalytic subunit PP2Ac, engaging multiple structural elements surrounding its active site. This interaction stabilizes the conformation of apo‐PP2Ac, prevents its aggregation, and is essential for acquiring phosphatase activity. In an ATP‐dependent manner, PTPA facilitates the recruitment of catalytic metal ions into the active site of PP2A and promotes ATP hydrolysis, both of which are crucial for the stable activation of the phosphatase. Thus, the formation of the PP2A–PTPA complex represents a key step in the functional maturation of PP2A [13].

Following the chemical derivatization of Cory and its conjugation with biotin, we performed affinity‐based protein pull‐down assays employing streptavidin‐coated magnetic beads. This approach is commonly employed for identifying and isolating proteins that specifically interact with modified small molecules. Due to their strong affinity for biotin, streptavidin‐coated beads enable efficient capture of proteins that bind to Cory‐Biotin conjugates within the sample. Using this approach, NQO1 was successfully identified as a direct binding target of Cory. NQO1, known as NAD(P)H:quinone oxidoreductase 1, is a cytoplasmic flavoenzyme that is highly overexpressed in multiple malignancies, including lung, breast, colorectal, pancreatic, ovarian, hepatic, and bladder cancers [14]. Elevated NQO1 expression in breast, lung, and cervical cancers is strongly associated with poor differentiation, advanced clinical stages, and lymphatic metastasis. Patients with high NQO1 expression show significantly reduced overall survival compared to those with low expression, suggesting an oncogenic role for NQO1 [15].

Despite recent findings, the precise mechanisms by which NQO1 contributes to tumorigenesis remain incompletely understood. NQO1 is a redox enzyme that protects cells from oxidative damage under conditions of oxidative stress [14]. Recent studies have shown that NQO1 interacts with proteins, including p33, p53, SIRT6, and eIF4G, thereby preventing their proteasomal degradation [16, 17, 18, 19]. In the present study, we have identified the interaction between NQO1 and PTPA, which may contribute to the suppression of PP2A activity in tumor cells with elevated NQO1 expression. This inhibition is associated with hyperactivation of signaling pathways, including PI3K/AKT and MAPK/ERK. Furthermore, we revealed the specific molecular mechanism by which Cory targets NQO1 and perturbs the NQO1–PTPA interface, leading to PP2A activation and subsequent suppression of the PI3K/AKT and MAPK/ERK signaling pathways, ultimately inhibiting tumor cell proliferation. These findings provide novel insights into the involvement of NQO1 in cancer progression, establishing a theoretical foundation for the development of therapeutic strategies targeting NQO1.

## 2 Results

### 2.1 Cory Exhibits Antiproliferative Activity Across Multiple Cancer Cell Lines by Activating PP2A

**FIGURE 1:**
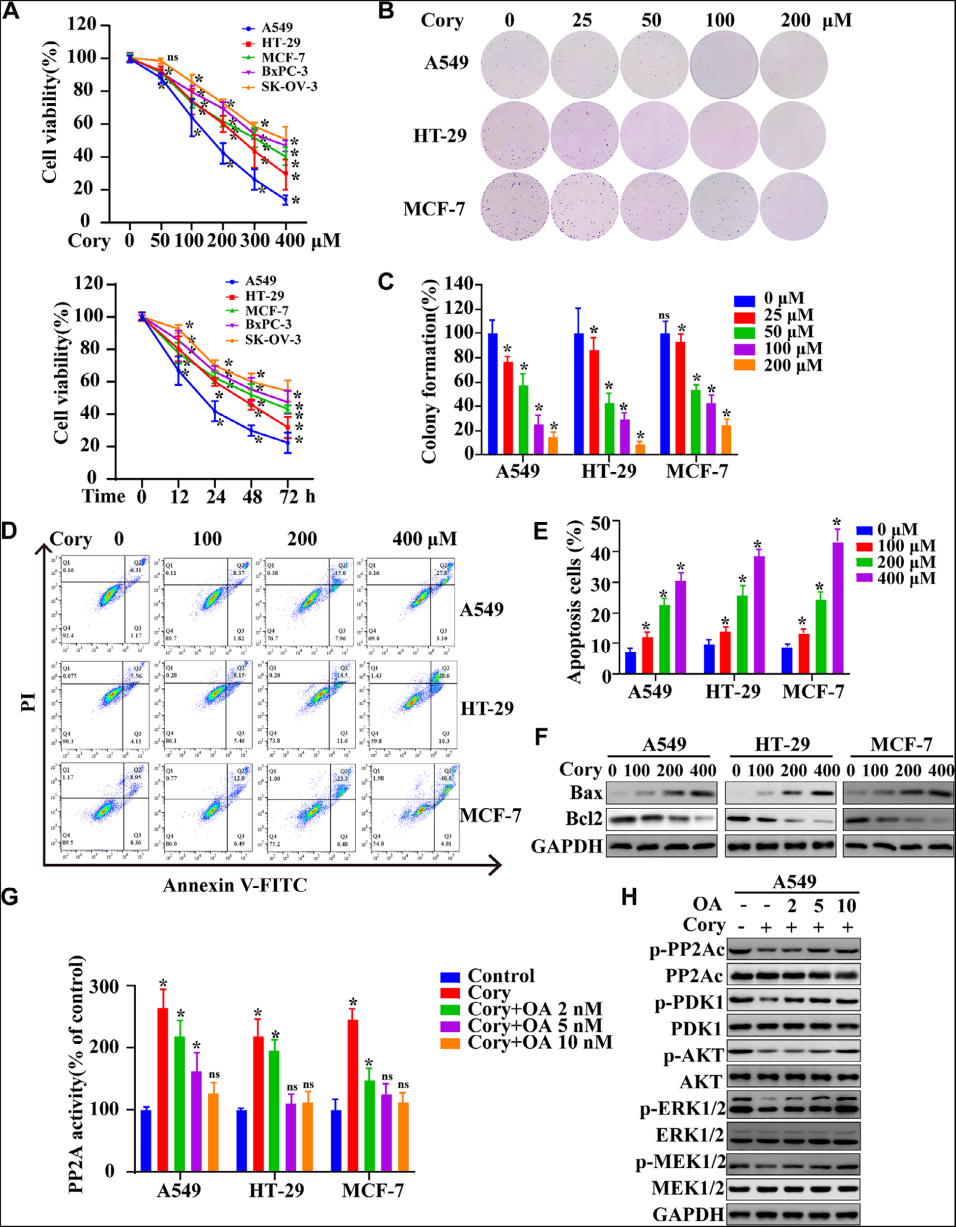
Cory exhibits anticancer activity in vitro. (A) Cell viability was assessed by CCK‐8 assay in A549, HT‐29, MCF‐7, BxPC‐3, and SK‐OV‐3 cells treated with Cory at the indicated concentrations (0–400 µM) for 24 h (upper panel) or with 200 µM Cory for the indicated times (0–72 h; lower panel). (B‐C) Colony formation assay in A549, HT‐29, and MCF‐7 cells treated with Cory (0–200 µM) for 24 h. Representative images (B) and quantitative analysis of relative colony numbers (C) are shown. (D‐E) Apoptosis was detected in A549, HT‐29, and MCF‐7 cells treated with Cory (0–400 µM) for 24 h. Representative images (D) and quantification of apoptotic cells (E) are presented. (F) Western blot analysis of Bax and Bcl2 expression in A549, HT‐29, and MCF‐7 cells treated with Cory (0–400 µM) for 24 h. (G) PP2A activity in A549, HT‐29, and MCF‐7 cells treated with 100 µM Cory alone or in combination with OA (0–10 nM) for 24 h. (H) Immunoblot analysis of phosphorylated and total PP2Ac, PDK1, AKT, ERK1/2, and MEK1/2 in A549 cells treated with 100 µM Cory alone or in combination with OA (0–10 nM) for 24 h. Data are presented as mean ± SD. Representative images of immunoblots are chosen from at least three independent experiments. Statistical differences were determined using an unpaired two‐tailed Student's t‐test for two‐group comparisons. For multiple comparisons, one‐way or two‐way ANOVA was applied, followed by Dunnett's post hoc test (for comparisons to a single control) or Tukey's post hoc test (for multiple pairwise comparisons); *P < 0.05; ns, not significant.

To evaluate the broad‐spectrum anticancer effects of Cory, A549 (human lung cancer) cells, HT‐29 (human colorectal cancer) cells, MCF‐7 (human breast cancer) cells, BxPC‐3 (human pancreatic cancer) cells, and SK‐OV‐3 (human ovarian cancer) cells were treated with varying concentrations of Cory for 24 h or with 200 µM Cory for 12, 24, 48, and 72 h. Cory reduced cell viability in a dose‐ and time‐dependent manner across these cell lines (Figure 1A). Colony formation assays revealed a marked reduction in the colony‐forming capacity following Cory treatment (Figures 1B,C and S1A,B). Annexin V‐FITC/PI staining was used to evaluate the effect of Cory on A549, HT‐29, and MCF‐7 cells, revealing a dose‐dependent induction of apoptosis (Figure 1D,E). This apoptotic phenotype was supported by increased Bax expression, reduced Bcl2 expression, PARP cleavage, and γ‐H2AX upregulation. Cleaved caspase‐3 was detected in caspase‐3‐expressing cells, whereas MCF‐7 cells, which lack endogenous caspase‐3, were evaluated using the remaining apoptosis‐associated markers (Figures 1F and S1C–E). Based on our previous findings that Cory activates PP2A in lung cancer, we investigated whether similar effects occur in colorectal and breast cancer cell lines. Treatment of A549, HT‐29, and MCF‐7 cells with Cory at specified concentrations significantly reduced phosphorylation of PP2Ac, indicating enhanced PP2A activity. Concurrently, decreased phosphorylation levels of downstream PP2A targets including PDK1, AKT, ERK1/2, and MEK1/2 were observed. These effects were effectively reversed by co‐treatment with the PP2A inhibitor okadaic acid (OA) in a dose‐dependent manner (Figures 1G,H and S1F,G). Furthermore, Cory treatment did not alter the protein expression levels of either PP2A A scaffold subunit (PP2Aa) or PP2A B regulatory subunit (PP2Ab) (Figure S1H,I). Collectively, these results demonstrate that Cory enhances PP2A activity by reducing PP2Ac phosphorylation, thereby suppressing the phosphorylation of key survival‐related kinases such as PDK1, AKT, ERK1/2, and MEK1/2.

**FIGURE 2:**
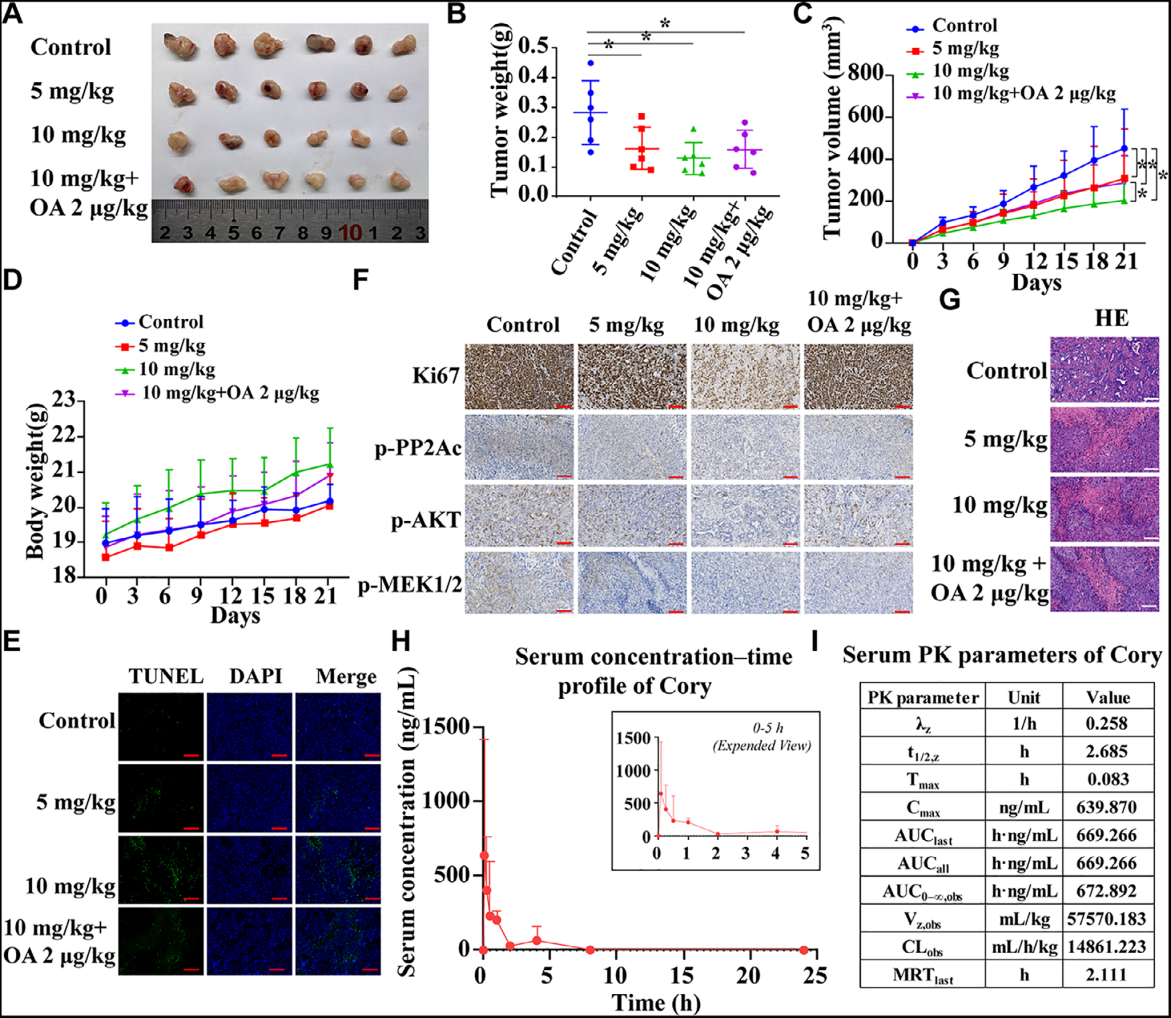
Cory suppresses tumor growth in vivo and shows its pharmacokinetic profile. (A–D) In vivo antitumor efficacy of Cory in A549 xenograft models. Gross images of excised tumors (A), tumor weight (B), tumor volume over time (C), and body weight changes (D) in mice treated with Cory (0, 5, or 10 mg/kg) or Cory (10 mg/kg) combined with okadaic acid (OA, 2 µg/kg) are shown (n = 6 mice per group). (E–G) TUNEL staining (E), IHC analysis of Ki67, p‐PP2Ac, p‐AKT, and p‐MEK1/2 (F), and H&E staining (G) were performed on tumor tissues selected from three mice per group. Representative staining images are shown. (H–I) Serum concentration–time profile of Cory (H) and derived pharmacokinetic parameters (I) following intravenous administration (10 mg/kg) in BALB/c nude mice. Serum concentrations were determined by LC‐MS/MS and pharmacokinetic parameters were calculated using non‐compartmental analysis. An expanded view of early time points (0–5 h) is shown for clarity. Scale bar = 100 µm; magnification: 20×. For panels A–D, data are presented as mean ± SD (n = 6 mice per group). For panels E–G, representative staining images are shown from three tumors per group. For panel H, serum concentrations are presented as mean ± SD (n = 3 mice per time point). Statistical significance for tumor‐growth experiments was determined using unpaired two‐tailed Student's t‐test or one‐way/two‐way ANOVA followed by Dunnett's or Tukey's post hoc test as appropriate; *P < 0.05; ns, not significant.

To evaluate the in vivo antitumor effects of Cory, a subcutaneous xenograft model was established using A549 cells. Mice were then treated with Cory (5 mg/kg or 10 mg/kg) alone or in combination with OA. Treatment with Cory significantly reduced tumor weight and volume in a dose‐dependent manner (Figure 2A–C). No obvious body weight loss was observed in Cory‐treated mice compared with control mice during the treatment period (Figure 2D). H&E staining of vital organs, including the heart, liver, spleen, lungs, and kidneys, showed no apparent toxicity (Figure S2A). TUNEL staining and Ki67 immunohistochemical (IHC) staining of tumor tissues indicated that Cory induced apoptosis and suppressed cell proliferation (Figures 2E,F and S2B,C). H&E staining also indicated histological lesions in tumor tissues following Cory treatment (Figure 2G). Furthermore, IHC staining was performed to examine the in situ expression of p‐PP2Ac, p‐AKT, and p‐MEK1/2. The results confirmed that Cory activated PP2A, thereby suppressing its downstream signaling targets (Figures 2F and S2C). Western blot analysis of tissue samples showed changes in the phosphorylation levels of PP2Ac, PDK1, AKT, ERK1/2, and MEK1/2, mirroring the cellular responses (Figure S2D,E). Co‐administration of Cory and OA partially reversed Cory‐induced tumor suppression in vivo (Figures 2A–G and S2A–E). We next evaluated the pharmacokinetic (PK) profile of Cory in mice. Following intravenous administration, Cory exhibited a serum *C_max_* of 639.870 ng/mL and an *AUC_last_* of 669.266 h·ng/mL (Figure 2H,I). Cory also displayed a large apparent volume of distribution (*V_z_* = 57.57 L/kg), consistent with extensive extravascular distribution. Together with the pharmacodynamic responses observed in tumor tissues, including PP2A activation and attenuation of downstream signaling pathways, these data support the in vivo activity of Cory. These findings demonstrate that Cory inhibits tumor growth in vivo by enhancing PP2A activity and attenuating its downstream signaling pathways.

### 2.2 Identification of NQO1 as a Direct Target of Cory

**FIGURE 3:**
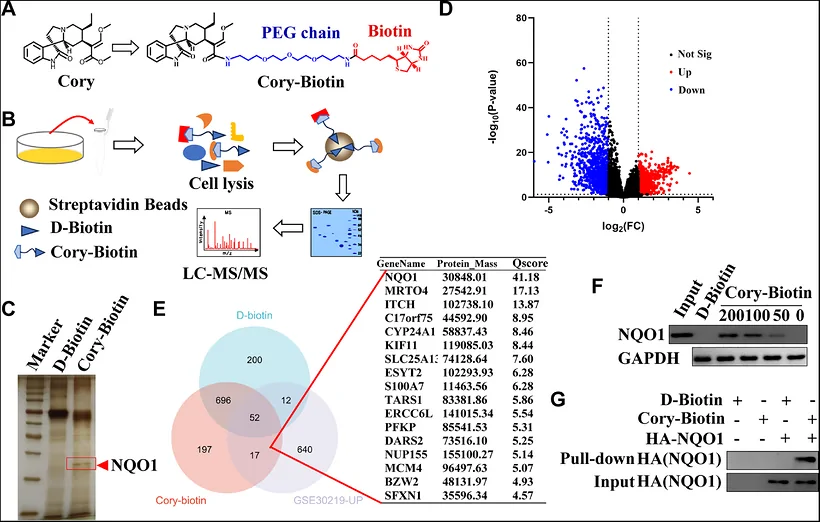
Identification of NQO1 as a direct target of Cory. (A) Chemical structures of Cory and biotin‐labeled Cory (Cory‐Biotin). (B) Schematic diagram of the protein fishing approach combined with mass spectrometry for identifying Cory‐Biotin‐binding proteins. (C) Silver‐stained SDS–PAGE analysis of proteins enriched from A549 whole‐cell lysates by D‐Biotin or Cory–Biotin pull‐down. Two parallel silver‐stained gels were prepared from the same pull‐down samples. The first gel was used to compare the protein profiles of the D‐Biotin and Cory–Biotin pull‐down samples, and the red box indicates the differential band region. In the second parallel gel, the corresponding differential band in the Cory–Biotin pull‐down lane was excised for in‐gel digestion and LC‐MS/MS analysis. (D) Volcano plot showing differentially expressed genes between lung cancer and adjacent normal tissues from the GEO dataset (GSE30219). (E) Venn diagram of proteins enriched in Cory‐Biotin pull‐down and proteins upregulated in lung cancer from GSE30219. Candidate overlapping proteins are listed. (F) A549 cell lysates were incubated with D‐Biotin or Cory‐Biotin (0–200 µM) for 8 h and then subjected to pull‐down with streptavidin beads, followed by Western blot analysis for NQO1. (G) Whole‐cell lysates from HEK 293T cells overexpressing HA‐NQO1 were incubated with D‐Biotin or Cory‐Biotin (100 µM) for 8 h, followed by pull‐down with streptavidin magnetic beads for 4 h. The precipitated complexes were then immunoblotted with anti‐HA antibody. Data are representative of three independent experiments.

Despite its promising antitumor efficacy, the direct molecular targets of Cory remain largely unknown. To identify its specific targets, we employed a protein fishing strategy using a biotin‐labeled derivative of Cory. Among various target identification methods, the protein fishing strategy using biotin‐labeled small molecules has emerged as a particularly powerful tool in biomedical research. Accordingly, a biotin moiety was conjugated to Cory to generate the Cory‐Biotin probe, which was verified by mass spectrometry (Figures 3A and S3A,B). We collected total protein lysates from A549 cells and incubated them with either Cory‐Biotin or D‐Biotin (control). Streptavidin magnetic bead‐based pull‐down assays were then performed (Figure 3B). Following trypsin digestion, proteins were identified by liquid chromatography–tandem mass spectrometry (LC‐MS/MS) (Figure 3C and Table S1). In parallel, we analyzed differentially expressed genes between lung cancer and adjacent normal tissues. The GSE30219 gene expression dataset from the GEO database was subjected to GEO2R analysis, and all differentially expressed genes with a false discovery rate (FDR) below 0.05 and |log_2_ FC| greater than 1 were included (Table S2). This yielded a collection of 721 upregulated genes (Figure 3D). Mass spectrometry data were further integrated with the Cory‐Biotin, D‐Biotin, and GSE30219‐UP datasets to generate a Venn diagram (Figure 3E, left). This strategy was designed to identify specific binding targets while minimizing false positives arising from non‐specific biotin interactions. Ultimately, 17 potential candidate proteins were identified (Figure 3E, right). Based on the molecular weights of significantly differentially expressed bands in the silver‐stained gel (Figure 3C, proteins highlighted in the red box), NQO1 was prioritized as a candidate Cory‐binding protein. To further confirm this result, mass spectrometry analysis was performed on the protein bands showing significant differences, as indicated by the red boxes (Table S3). Quantitative analysis of relative protein abundance provided further evidence supporting the identification of NQO1. To validate the interaction, pull‐down assays were performed using Cory‐Biotin at various concentrations in A549 cells. Western blot (WB) results showed a concentration‐dependent enrichment of NQO1 (Figure 3F). Additionally, Cory‐Biotin successfully pulled down exogenously overexpressed NQO1 from 293T cells (Figure 3G). Together, these findings provide preliminary validation for NQO1 as a potential target of Cory.

### 2.3 Validation of Cory Interaction with NQO1

**FIGURE 4:**
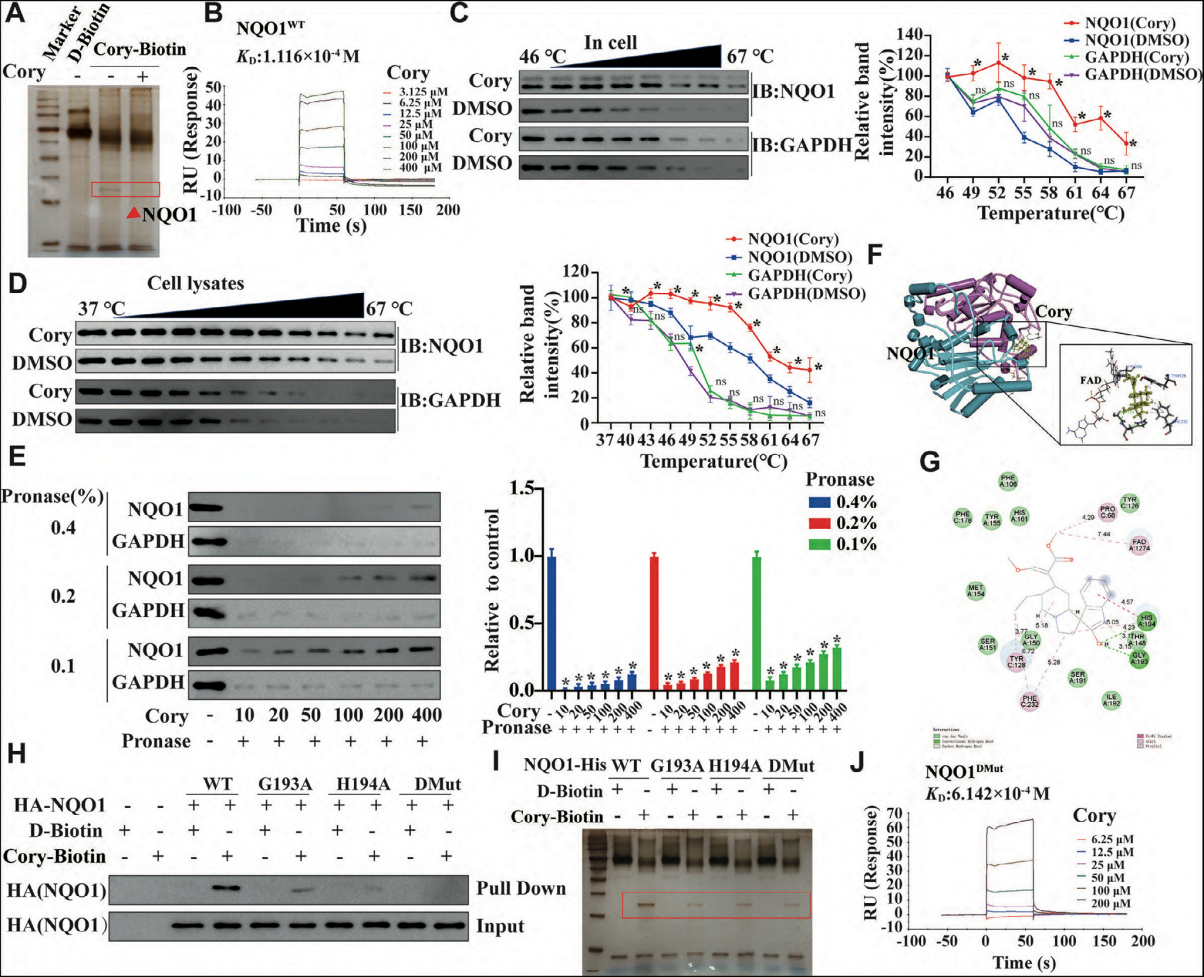
Validation of Cory interaction with NQO1. (A) Pull‐down assay using streptavidin magnetic beads. A549 cell lysates were divided into three groups: D‐Biotin, Cory‐Biotin, and Cory‐Biotin pre‐incubated with 100 µM Cory for 6 h. Each group was incubated with lysates at 4 °C for 8 h, followed by bead incubation for 4 h. Precipitated proteins were detected by silver staining. (B) SPR sensorgrams showing the binding affinity between purified NQO1 protein and Cory. (C) CETSA of A549 cells treated with Cory (100 µM) or DMSO for 8 h. Total proteins were extracted and subjected to heating at indicated temperatures (46–67 °C). Relative NQO1 levels were quantified. (D) CETSA of A549 cell‐derived proteins incubated with Cory (100 µM) or DMSO for 4 h, followed by heating (37–67°C). Relative NQO1 levels are shown. (E) DARTS assay demonstrating Cory‐induced stabilization of NQO1. A549 lysates were incubated with Cory or vehicle for 1 h at room temperature, followed by pronase digestion at indicated ratios for 20 min. NQO1 levels were analyzed by immunoblotting. (F) Predicted three‐dimensional structure of the NQO1‐Cory complex. (G) Two‐dimensional interaction diagram between NQO1 and Cory. (H) Pull‐down assay using streptavidin beads with lysates from HEK 293T cells transfected with HA‐tagged wild‐type (WT) or mutant (G193A, H194A, or double mutant G193A/H194A) NQO1. Lysates were incubated with Cory‐Biotin or D‐Biotin for 8 h. Bound proteins (Pull‐down) and total inputs were detected by anti‐HA immunoblotting. (I) Pull‐down of purified 6×His‐tagged WT or mutant NQO1 proteins incubated with Cory‐Biotin or D‐Biotin for 8 h. Precipitated proteins were detected by silver staining. (J) SPR analysis of the binding between Cory and the NQO1 double mutant (G193A/H194A). Data are presented as mean ± SD from three independent biological replicates. Representative images are shown from at least three independent experiments. Statistical significance between two groups was determined using an unpaired two‐tailed Student's t‐test, while comparisons among multiple groups were analyzed using one‐way or two‐way ANOVA followed by Dunnett's post hoc test; *P < 0.05.

To further validate the interaction between Cory and NQO1, A549 cell lysates were pre‐incubated with free Cory molecule, followed by a pull‐down assay using Cory‐Biotin. Notably, pre‐incubation with Cory markedly reduced the binding of Cory‐Biotin to NQO1 compared to the non‐pretreated control (Figure 4A, proteins highlighted in the red box). Surface plasmon resonance (SPR) analysis determined an equilibrium dissociation constant (*K*_D_) of 1.116 × 10^−4^ M for the Cory–NQO1 interaction, indicating that Cory binds NQO1 with moderate micromolar‐range affinity (Figure 4B). The cellular thermal shift assay (CETSA) is based on the principle that protein thermal stability increases upon ligand binding [20]. Pre‐incubation of Cory either in live cells or co‐incubation with cell lysates demonstrated the effective protection of NQO1 from temperature‐dependent degradation (Figure 4C,D), and this protective effect was concentration‐dependent at specific temperatures (Figure S4A). Drug affinity responsive target stability (DARTS) relies on the ability of a drug‐target protein binding to confer resistance to protease digestion on the target protein [21]. Our results indicated that Cory concentration‐dependently inhibits the degradation of NQO1 induced by pronase (Figure 4E).

Molecular docking studies based on the crystal structure of human NQO1 protein, sourced from the PDB database (http://www.rcsb.org ID: 1H69) revealed that NQO1 exists as a dimer of two identical monomers (designated as A and C) in complex with an FAD cofactor. The ligand binding site is located within a pocket adjacent to the FAD‐containing region, including Tyr128, Tyr126, Trp105, Pro68, Phe232, and the FAD cofactor. Cory is predicted to associate with NQO1 through a network of hydrogen bonds, hydrophobic contacts, and van der Waals interactions (Figures 4F and S4B,C). Hydrogen bonds are formed between Cory and Gly193 and His194, while van der Waals interactions were observed with Phe106, Tyr126, Thr148, Gly150, Ser151, Met154, Tyr155, His161, Phe178, Ser191, and Ile192. Additionally, Cory engaged in hydrophobic interactions with Pro68, Tyr128, His194, and Phe232 (Figure 4G). To evaluate the contribution of Gly193 and His194 to Cory recognition, these residues were individually or jointly mutated to alanine (referred to as NQO1‐G193A, NQO1‐H194A, and NQO1‐DMut), and recombinant NQO1 proteins were expressed in 293T cells or prokaryotic expression systems (Figure S4D–G). Pull‐down assays revealed reduced, but not abolished, Cory binding in both single and double mutations (Figures 4H,I and S4H,I). SPR analysis further validated the interaction between Cory and NQO1‐DMut, revealing a *K*_D_ value of 6.142 × 10^−4^ M, indicating that G193A/H194A mutation moderately decreases Cory–NQO1 binding affinity (Figure 4J). The residual binding observed for NQO1‐DMut suggests that additional surrounding hydrophobic and van der Waals interactions also contribute to Cory recognition. Under physiological conditions, NQO1 plays a crucial role in scavenging superoxide radicals, maintaining cellular redox balance, and preserving cellular homeostasis [14]. Notably, Cory treatment did not significantly affect NQO1 enzymatic activity in either A549 cell lysates or purified recombinant NQO1, nor did it increase intracellular reactive oxygen species levels (Figure S4J–L). In summary, these findings suggest that Cory directly interacts with NQO1, with Gly193 and His194 serving as important contributing residues for Cory recognition.

### 2.4 Cory Activates PP2A by Attenuating the NQO1–PTPA Interaction

**FIGURE 5:**
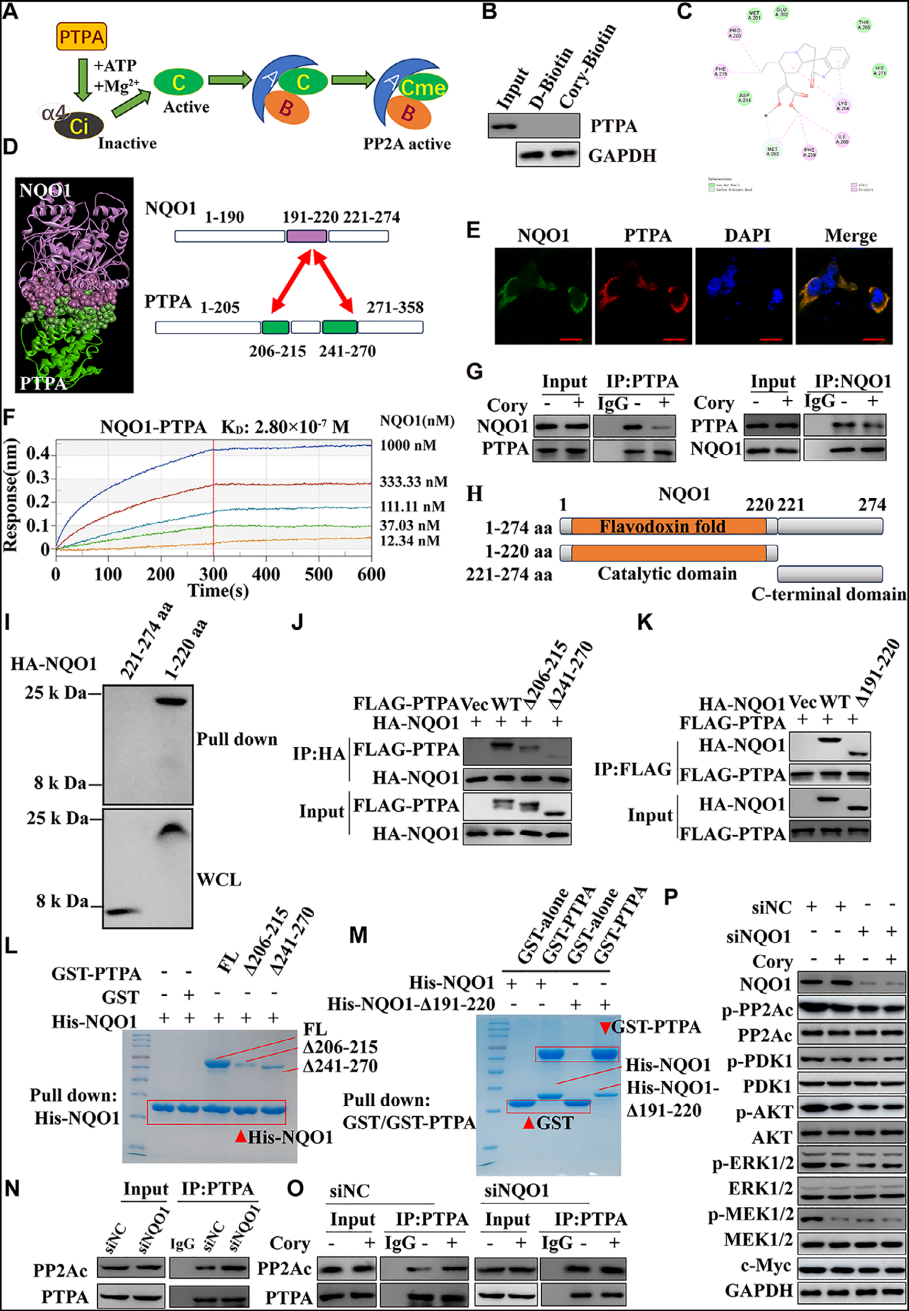
Cory activates PP2A by attenuating the NQO1–PTPA interaction. (A) Schematic diagram of PTPA‐mediated activation of PP2A. (B) Pull‐down assay using streptavidin beads with A549 whole‐cell lysates incubated with D‐Biotin or Cory‐Biotin for 8 h. Precipitated proteins were immunoblotted for PTPA. (C) Exploratory docking analysis of Cory with PTPA. The model did not reveal obvious hydrogen‐bond interactions or a favorable interaction pattern, which is consistent with the pull‐down result showing no detectable direct Cory–PTPA binding. (D) Computational docking model of the NQO1–PTPA interaction. (E) Confocal microscopy images showing co‐localization of NQO1 and PTPA. (F) BLI analysis of the direct interaction between purified NQO1 and PTPA. Real‐time binding and dissociation sensorgrams were recorded with immobilized PTPA and varying concentrations of NQO1. (G) Co‐immunoprecipitation (Co‐IP) of NQO1 and PTPA in A549 cells treated with Cory (0 or 100 µM) for 24 h. (H) Domain architecture of NQO1. (I) Pull‐down assay using Cory‐Biotin (100 µM, 8 h) and streptavidin beads with lysates of HEK 293T cells expressing HA‐tagged NQO1(1–220) or NQO1(221–274). Precipitated proteins were immunoblotted with anti‐HA antibody. (J) Co‐IP assay of interactions between HA‐NQO1 and FLAG‐PTPA, FLAG‐PTPA‐Δ206–215, or FLAG‐PTPA‐Δ241–270. (K) Co‐IP assay of interactions between FLAG‐PTPA and HA‐NQO1 or HA‐NQO1‐Δ191–220. (L) His pull‐down assay using His‐NQO1 bound to Ni‐NTA beads incubated with GST, GST‐PTPA, GST‐PTPA‐Δ206–215, or GST‐PTPA‐Δ241–270. Bound proteins were detected by Coomassie Blue staining. (M) GST pull‐down assay using GST or GST‐PTPA bound to glutathione beads incubated with purified NQO1 or NQO1‐Δ191–220. Bound proteins were detected by Coomassie Blue staining. (N) Co‐IP of endogenous PTPA and PP2Ac in A549 cells transfected with siNC or siNQO1. (O) Co‐IP of PTPA and PP2Ac in A549 cells transfected with siNC or siNQO1 and treated with Cory (0 or 100 µM) for 24 h. (P) Western blot analysis of NQO1, PP2Ac, PDK1, AKT, MEK1/2, ERK1/2, and c‐Myc in A549 cells transfected with siNC or siNQO1 and treated with Cory (0 or 100 µM) for 24 h. Data are representative of three independent experiments. Scale bar: 10 µM.

PP2A activity is tightly regulated by PTPA (Figure 5A). Here we demonstrate that Cory activates PP2A, leading to subsequent inhibition of downstream effectors including PDK1, AKT, ERK1/2, and MEK1/2 (Figure 1). This activation may represent a key mechanism underlying its antitumor activity. However, the specific molecular mechanism through which Cory activates PP2A remains unclear. Biotin‐based pull‐down assays showed that Cory‐Biotin did not enrich PTPA from A549 whole‐cell lysates (Figure 5B). Consistently, exploratory docking analysis did not predict stable hydrogen‐bond interactions between Cory and PTPA, suggesting a lack of favorable direct Cory–PTPA binding under the tested conditions (Figure 5C). These results indicate that Cory‐induced PP2A activation is unlikely to result from direct binding of Cory to PTPA. Additionally, Cory treatment in A549, HT‐29, and MCF‐7 cells did not significantly alter the protein or mRNA levels of NQO1 or PTPA (Figure S5A–C). Structure‐based docking using the CDOCKER algorithm revealed a potential binding interface between NQO1 and PTPA [22]. Docking analysis suggests that residues 191 to 220 of NQO1 are involved in interaction with PTPA domains spanning residues 206‐215 and 241‐270 (Figure 5D). Confocal imaging confirmed the co‐localization of NQO1 and PTPA in cells (Figure 5E). Biolayer interferometry (BLI) further confirmed a direct physical interaction with submicromolar affinity between purified NQO1 and PTPA in vitro, yielding a *K*_D_ of 2.80 × 10^−7^ M (Figure 5F). Considering that this affinity is higher than the Cory–NQO1 affinity measured by SPR, we interpret Cory as an NQO1‐dependent interface modulator rather than a classical high‐affinity competitive ligand. Consistently, Co‐IP assays revealed a direct interaction between NQO1 and PTPA, which was disrupted upon Cory treatment (Figures 5G and S5D,E). NQO1 consists of a catalytic domain (peptide segment 1‐220) and a C‐terminal domain (peptide segment 221‐274). Overexpression of HA‐NQO1 (1‐220) and HA‐NQO1 (221‐274) fragments in 293T cells, followed by pull‐down assays with Cory‐Biotin and streptavidin beads, indicated that Cory is associated with the catalytic domain of NQO1 (Figure 5H,I). Similarly, pull‐down assays using wild‐type and truncated forms of NQO1 or PTPA, expressed in 293T cells or prokaryotic expression systems, revealed a significant reduction in the interaction between truncated PTPA (Δ206‐215 or Δ241‐270) and NQO1, as well as between truncated NQO1 (Δ191‐220) and PTPA (Figures 5J–M and S5F–N). PTPA promotes PP2A activation by binding to the PP2Ac subunit, altering its catalytic conformation, promoting PP2A assembly, and enhancing its catalytic activity. This physical interaction represents a crucial step in mediating PTPA‐induced PP2A activation [13]. Because PTPA is an essential activator of PP2A, Cory‐induced weakening of the NQO1–PTPA interface may facilitate the redistribution of PTPA toward PP2Ac, thereby promoting PP2A holoenzyme assembly and catalytic activation. To preliminarily validate whether the NQO1 interaction influences PTPA–PP2Ac binding, we transiently silenced NQO1 in A549 cells using siRNA targeting NQO1 (siNQO1). Compared with siNC‐treated cells, NQO1 depletion enhanced the interaction between PTPA and PP2Ac (Figures 5N and S5O). Remarkably, Cory enhanced PTPA–PP2Ac interaction only in the siNC group, but not in the siNQO1 group (Figures 5O and S5P). Following NQO1 knockdown, Cory failed to decrease PP2Ac phosphorylation (p‐PP2Ac), indicating that Cory‐induced PP2A activation was largely abolished and the phosphorylation levels of PDK1, AKT, ERK1/2, and MEK1/2, as well as c‐Myc expression, remained unchanged (Figure 5P; Figures S5Q and S5R). Previous studies also suggested that NQO1 depletion enhances PP2A activity, consistent with our findings [11]. Collectively, these findings suggest that Cory disrupts NQO1–PTPA interaction, thereby promoting PP2A activation and suppressing downstream signaling.

### 2.5 High Expression of NQO1 Contributes to Tumorigenesis

**FIGURE 6:**
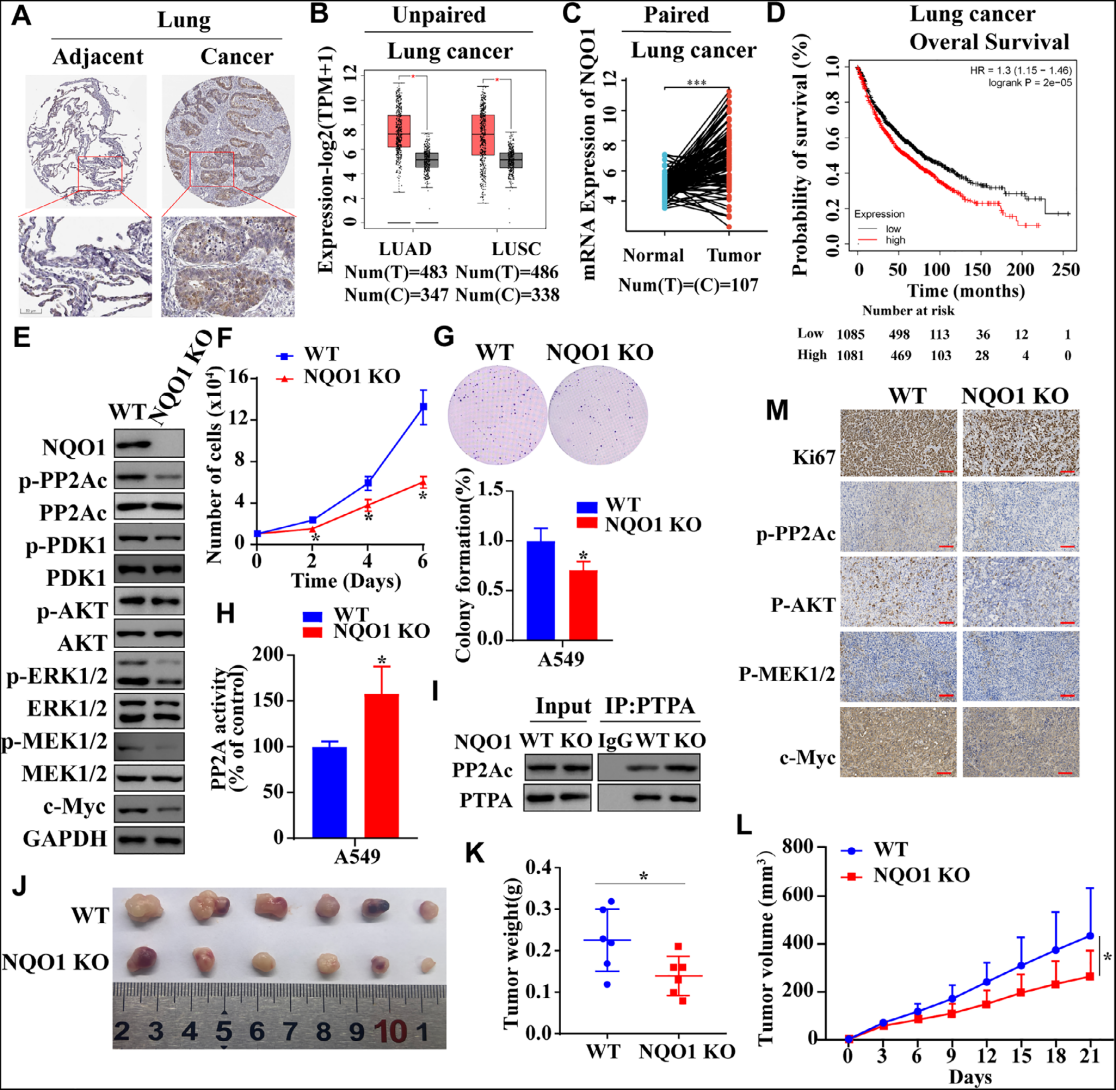
High expression of NQO1 contributes to tumorigenesis. (A) Representative immunohistochemical staining images showing NQO1 protein expression in adjacent normal lung tissue and lung cancer tissue from human tissue microarrays. Images were obtained from the Human Protein Atlas; lower panels show magnified views of the boxed regions. (B) Unpaired analysis of *NQO1* mRNA expression in lung cancer tissues and normal lung tissues from the TCGA/GTEx dataset. NQO1 expression was significantly increased in both lung adenocarcinoma (LUAD; tumor, n = 483; normal, n = 347) and lung squamous cell carcinoma (LUSC; tumor, n = 486; normal, n = 338). (C) Paired analysis of *NQO1* mRNA expression in lung tumor tissues and matched adjacent non‐tumor tissues from the TCGA dataset (n = 107 pairs). (D) Kaplan–Meier survival analysis of the association between NQO1 expression and overall survival in lung cancer patients using the Kaplan–Meier Plotter database. (E) Western blot analysis of NQO1, PP2Ac, PDK1, AKT, MEK1/2, ERK1/2, and c‐Myc in wild‐type (WT) and NQO1 knockout (KO) A549 cells generated by CRISPR/Cas9. (F) Growth curves of WT and NQO1 KO A549 cells. (G) Colony formation assay of WT and NQO1 KO A549 cells. (H) PP2A activity in WT and NQO1 KO A549 cells. (I) Co‐IP of the interaction between PTPA and PP2Ac in WT and NQO1 KO A549 cells. (J) Representative images of xenograft tumors derived from WT and NQO1 KO A549 cells. (K) Tumor weight in xenograft models. (L) Tumor volume changes in xenograft models. (M) IHC analysis of Ki67, p‐PP2Ac, p‐AKT, p‐MEK1/2, and c‐Myc in xenograft tumor sections. Scale bar: 100 µM; magnification: ×20. Data are presented as mean ± SD from three independent biological replicates for in vitro experiments or from six mice per group for in vivo experiments. Representative immunoblotting and histological images are shown from at least three independent experiments. Statistical significance between two independent groups was determined using an unpaired two‐tailed Student's t‐test, while paired clinical samples were analyzed using a paired t‐test. Survival analysis was evaluated using the log‐rank test; *P < 0.05.

The marked elevation of NQO1 protein expression in tumor tissues of lung, breast, and colorectal cancers, relative to adjacent normal tissues, is consistent with data from the Human Protein Atlas (Figures 6A and S6A). GEPIA analysis based on TCGA and GTEx datasets further confirmed significant upregulation of *NQO1* mRNA in tumor tissues of lung, breast, and colorectal cancers (Figures 6B,C and S6B,C). Elevated NQO1 expression has been associated with poorer survival outcomes in patients with lung, breast, and colorectal cancers (Figures 6D and S6D). Collectively, these data suggest that NQO1 contributes to tumorigenesis and may serve as a prognostic biomarker. To evaluate the role of NQO1 in tumor growth, NQO1 was depleted in A549 cells using CRISPR/Cas9 technology. Immunoblotting confirmed efficient knockout of NQO1 in A549 cells (Figures 6E and S6E). NQO1 deletion significantly suppressed cell proliferation and viability while enhancing intracellular ROS levels (Figures 6F,G and S6F). NQO1 depletion reduced inhibitory PP2Ac phosphorylation and enhanced overall PP2A activity, thereby reducing phosphorylation of downstream PDK1, AKT, MEK1/2, and ERK1/2 and downregulating c‐Myc expression (Figures 6E,H and S6E). Compared to wild‐type cells, NQO1‐knockout A549 cells showed increased interaction between PTPA and PP2Ac (Figures 6I and S6G). In xenograft models, NQO1 loss significantly suppressed tumor growth (Figure 6J–L). Reduced Ki67 positivity indicated impaired proliferation of A549 cells in vivo upon NQO1 deletion (Figures 6M and S6H). The IHC staining results demonstrate that NQO1 loss significantly reduced p‐PP2Ac, p‐AKT, p‐MEK1/2, and c‐Myc staining intensities (Figures 6M and S6H). Additionally, protein‐level changes in tumor tissues mirrored the cellular findings (Figure S6I,J). These findings highlight the role of NQO1 in supporting tumor cell proliferation and survival.

### 2.6 Cory Exerts Antitumor Effects in an NQO1‐Dependent Manner

**FIGURE 7:**
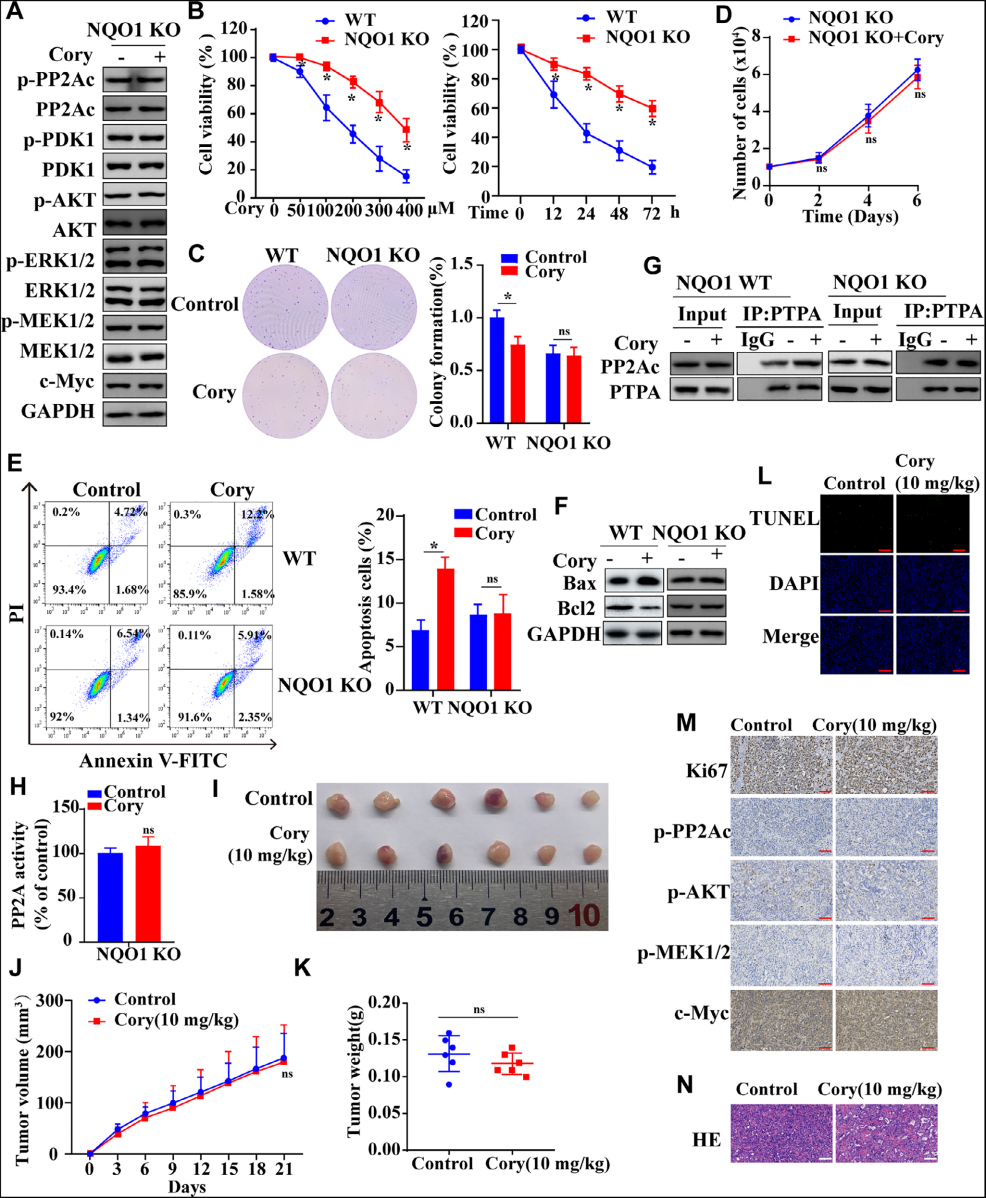
Cory exerts antitumor effects in an NQO1‐dependent manner. (A) Western blot analysis of NQO1, PP2Ac, PDK1, AKT, MEK1/2, ERK1/2, and c‐Myc in A549 NQO1 KO cells treated with Cory (0 or 100 µM) for 24 h. (B) Cell viability of WT and NQO1 KO A549 cells treated with Cory (0–400 µM) for 24 h (left) or with 200 µM Cory for 12–72 h (right), assessed by CCK‐8 assay. (C) Colony formation assay of WT and NQO1 KO A549 cells treated with Cory (0 or 100 µM) for 24 h. The histogram shows the percentage of colony numbers relative to the control. (D) Growth curves of A549 NQO1 KO cells treated with Cory (0 or 100 µM) for 24 h; cell numbers were monitored for 6 days. (E) Apoptosis assay of WT and NQO1 KO A549 cells treated with Cory (0 or 100 µM) for 24 h. Representative images (left) and quantitative analysis (right) are shown. (F) Western blot analysis of Bax and Bcl2 in WT and NQO1 KO A549 cells treated with Cory (0 or 100 µM) for 24 h. (G) Co‐IP of the PTPA–PP2Ac interaction in WT and NQO1 KO A549 cells treated with Cory (0 or 100 µM) for 24 h. (H) PP2A activity in A549 NQO1 KO cells treated with Cory (0 or 100 µM) for 24 h. (I‐K) Antitumor efficacy of Cory (10 mg/kg) in mice bearing A549 NQO1 KO cell xenografts: representative tumor images (I), tumor volume curves (J), and tumor weight (K). (L) TUNEL staining of tumor tissues. (M) IHC analysis of Ki67, p‐PP2Ac, p‐AKT, p‐MEK1/2, and c‐Myc in xenograft tumors. (N) H&E staining of tumor sections. Scale bar: 100 µM; magnification: ×20. Data are presented as mean ± SD from three independent biological replicates for in vitro experiments or from six mice per group for in vivo experiments. Representative images are shown from at least three independent experiments. Statistical significance between two independent groups was determined using an unpaired two‐tailed Student's t‐test. Time‐course and concentration‐response data were analyzed using two‐way ANOVA. Other comparisons among multiple groups were analyzed using one‐way ANOVA followed by Tukey's post hoc test; *P < 0.05; ns, not significant.

To investigate the dependency of Cory‐mediated tumor suppression on NQO1, we treated A549 NQO1 KO cells with Cory. NQO1 deletion abolished Cory‐induced enhancement of PP2A activity and the subsequent downregulation of phosphorylated PDK1, AKT, ERK1/2, and MEK1/2, as well as c‐Myc expression (Figures 7A,H and S7A). Knocking out NQO1 moderately attenuated the inhibitory effect of Cory on cell proliferation (Figure 7B). After NQO1 knockout, Cory treatment did not significantly alter clonogenic capacity, cell proliferation rate, or apoptosis compared to the control group (Figures 7C–F and S7B). Moreover, Cory treatment did not affect the interaction between PTPA and PP2Ac in A549 NQO1 KO cells (Figures 7G and S7C). Similarly, Cory exhibited a negligible effect on PP2A activity (Figure 7H). In a subcutaneous tumor model established using A549 NQO1 KO cells, Cory failed to exert a significant inhibitory effect on tumor growth (Figure 7I–K). Ki67, TUNEL, and H&E staining demonstrated no significant variations in proliferation, apoptosis, or tissue necrosis (Figures 7L–N and S7D,E). IHC analysis of p‐PP2Ac, p‐AKT, p‐MEK1/2, and c‐Myc showed no notable differences in expression following Cory treatment (Figures 7M and S7E). Tissue protein expression analysis of PP2Ac, PDK1, AKT, ERK1/2, MEK1/2, and c‐Myc indicated no significant differences between the Cory‐treated group and the control group (Figure S7F,G). In summary, these findings support the conclusion that the antitumor effect of Cory is NQO1‐ dependent.

### 2.7 Gly193 and His194 of NQO1 are Functionally Required for Cory‐Mediated Anticancer Activity

**FIGURE 8:**
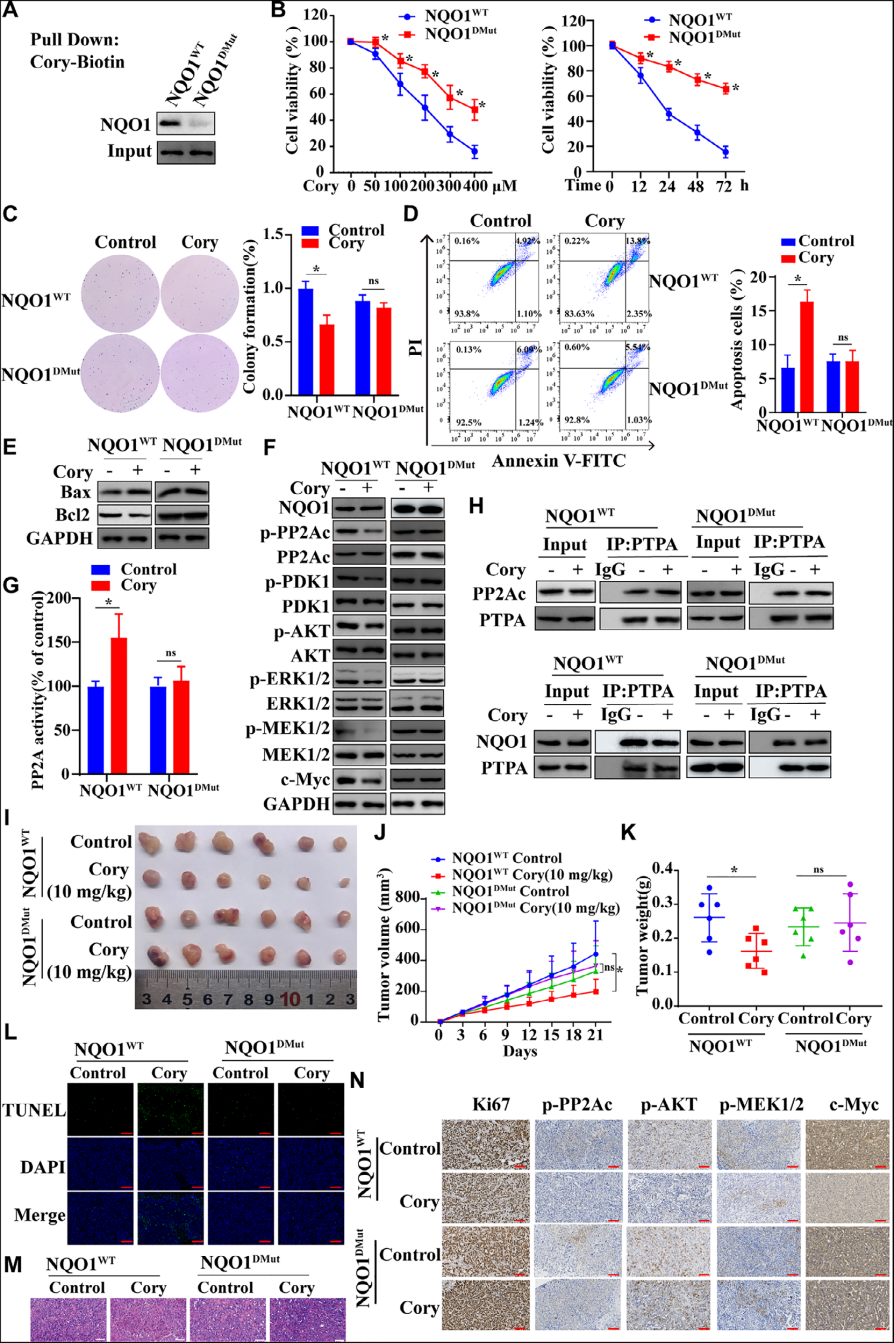
Gly193 and His194 of NQO1 are required for the anticancer activity of Cory. (A) Pull‐down assay using streptavidin beads. Lysates from A549 cells expressing NQO1^WT^ and NQO1^DMut^ (G193A/H194A) were incubated with Cory‐Biotin for 8 h. Precipitated NQO1 was detected by Western blot. (B) Cell viability of NQO1^WT^ and NQO1^DMut^ A549 cells treated with Cory (0–400 µM) for 24 h (left) or with 200 µM Cory for 12–72 h (right), assessed by CCK‐8 assay. (C) Colony formation assay of NQO1^WT^ and NQO1^DMut^ A549 cells treated with Cory (0 or 100 µM) for 24 h. The histogram shows the percentage of colony numbers relative to the control. (D) Apoptosis assay of NQO1^WT^ and NQO1^DMut^ A549 cells treated with Cory (0 or 100 µM) for 24 h. Representative images and quantitative analysis are shown. (E) Western blot analysis of Bax and Bcl2 in NQO1^WT^ and NQO1^DMut^ A549 cells treated with Cory (0 or 100 µM) for 24 h. (F) Western blot analysis of NQO1, PP2Ac, PDK1, AKT, MEK1/2, ERK1/2, and c‐Myc in NQO1^WT^ and NQO1^DMut^ A549 cells treated with Cory (0 or 100 µM) for 24 h. (G) PP2A activity in NQO1^WT^ and NQO1^DMut^ A549 cells treated with Cory (0 or 100 µM) for 24 h. (H) Co‐IP assays of PTPA interactions with PP2Ac and NQO1 in NQO1^WT^ and NQO1^DMut^ A549 cells treated with Cory (0 or 100 µM) for 24 h. (I‐K) Antitumor efficacy of Cory (10 mg/kg) in mice bearing NQO1^WT^ and NQO1^DMut^ A549 cell xenografts: representative tumor images (I), tumor volume curves (J), and tumor weight (K). (L) TUNEL staining of tumor tissues. (M) H&E staining of tumor sections. (N) IHC analysis of Ki67, p‐PP2Ac, p‐AKT, p‐MEK1/2, and c‐Myc in xenograft tumors. Scale bar: 100 µM; magnification: ×20. Data are presented as mean ± SD from three independent biological replicates for in vitro experiments or from six mice per group for in vivo experiments. Representative images are shown from at least three independent experiments. Statistical significance between two independent groups was determined using an unpaired two‐tailed Student's t‐test. Time‐course and concentration‐response data were analyzed using two‐way ANOVA. Other comparisons among multiple groups were analyzed using one‐way ANOVA followed by Tukey's post hoc test; *P < 0.05; ns, not significant.

Stable cell lines expressing either wild‐type NQO1 (NQO1^WT^) or double‐mutant NQO1 (NQO1^DMut^) were established in A549 NQO1 KO cells using lentiviral‐mediated gene transfection. Cory‐Biotin pull‐down revealed a significant reduction in the binding affinity between re‐expressed NQO1^DMut^ and Cory (Figures 8A and S8A). Compared with the NQO1^WT^ cell line, Cory exhibited a diminished anti‐proliferative effect in the NQO1^DMut^ cell line, indicating that G193A/H194A mutation impairs the functional response to Cory (Figure 8B). Similarly, Cory had no significant effect on cell viability or apoptosis in NQO1^DMut^ cell line (Figures 8C–E and S8B). In NQO1^DMut^ cells, Cory failed to activate PP2A and inhibit downstream targets, including PDK1, AKT, ERK1/2, MEK1/2, and c‐Myc expression (Figures 8F,G and S8C). Furthermore, Cory did not affect the interactions between NQO1 and PTPA or between PTPA and PP2Ac in the NQO1^DMut^ cell line (Figures 8H and S8D). To further elucidate the role of NQO1 Gly193 and His194 sites in Cory's in vivo antitumor effects, xenograft models were established using cells expressing either NQO1^WT^ or NQO1^DMut^. Cory suppressed tumor growth in the NQO1^WT^ xenograft model, but showed no significant effect in the NQO1^DMut^ tumor model (Figure 8I–K). Ki67, TUNEL, and H&E staining demonstrated that Cory failed to suppress proliferation or induce apoptosis in the NQO1^DMut^ tumor model (Figures 8L–N and S8E,F). Cory decreased the positive staining for p‐PP2Ac, p‐AKT, p‐MEK1/2, and c‐Myc in NQO1^WT^ tumors, whereas this response was abolished in NQO1^DMut^ tumors (Figures 8N and S8F). Furthermore, Cory enhanced PP2A activity and reduced the levels of p‐PP2Ac, p‐PDK1, p‐AKT, p‐MEK1/2, and c‐Myc expression only in the NQO1^WT^ model (Figure S8G,H). In summary, the antitumor efficacy of Cory was nearly abolished in the A549 NQO1^DMut^ xenograft model, suggesting that Gly193 and His194 are functionally critical for coupling Cory binding to modulation of the PTPA –NQO1/PP2A switch in vivo.

## 3 Discussion

The precise coordination of kinase and phosphatase activities is a fundamental mechanism regulating cellular signaling, and its dysregulation is a cornerstone of oncogenic transformation. As critical hubs in signaling networks, protein kinases are frequently aberrantly activated in cancer, highlighting the essential counter‐regulatory role of phosphatases such as PP2A [23]. Malignant tumors are characterized by hallmarks including sustained proliferative signaling, evasion of cell death, induction of angiogenesis, and enhanced invasive and migratory capabilities [24]. PP2A acts as a critical regulatory node that integrates the PI3K/AKT and MAPK/ERK signaling cascades [11]. Although the structural complexity and intricate regulation of heterotrimeric PP2A present challenges for its direct targeting, compelling therapeutic strategies have emerged that reactivate PP2A pharmacologically by inhibiting its endogenous suppressors. This paradigm is further supported by evidence that metformin, paeonol, and resveratrol share a convergent mechanism of PP2A activation to suppress tumor growth, invasion, chemoresistance, and adhesion [25, 26, 27]. Together, these findings highlight both the utility and complexity of targeting PP2A in cancer treatment.

From a pharmacological perspective, restoring the tumor‐suppressive activity of PP2A using small‐molecule activators has emerged as a promising therapeutic strategy in oncology. FTY720, the prototypical PP2A activator originally developed as an immunomodulator, enhances holoenzyme activity to blunt oncogenic AKT and ERK signaling cascades [7, 28]. However, these off‐target interactions with S1P receptors and PKC inevitably provoke severe systemic adverse outcomes, including immunosuppression and bradycardia, substantially narrowing its clinical utility [29, 30]. Subsequent structural innovations led to small molecules such as SMAPs, which drive allosteric conformational changes by directly binding to the PP2Aα scaffold subunit, and indirect activators like niclosamide that rescue PP2A function by dismantling its endogenous inhibitor CIP2A [31, 32]. Distinct from broad‐spectrum PP2A activators, Cory functions via a context‐dependent mechanism by specifically uncoupling the NQO1–PTPA complex. Furthermore, the loss of Cory‐induced PP2A activation in NQO1‐deficient models indicates that NQO1 is essential for this response. Together with the interaction data, these findings establish the NQO1–PTPA regulatory axis as a major mechanism underlying Cory‐mediated PP2A activation. Targeting this NQO1‐driven oncogenic interaction, rather than globally altering PP2A assembly, may reduce typical off‐target effects. These findings support Cory as a potentially selective lead compound with a favorable preliminary safety profile for NQO1‐overexpressing cancers.

Our previous work identified Cory, a natural small molecule, as a potent activator of PP2A. However, the precise mechanism of Cory‐induced PP2A activation remained unclear. Using protein pull‐down assays, mass spectrometry, CETSA, DARTS technology, and SPR, we successfully identified NQO1 as a direct target of Cory. Physiologically, NQO1 is expressed at low levels in various normal tissues, where it functions in quinone detoxification, superoxide clearance, and stabilizing regulatory proteins [14]. Notably, NQO1 is aberrantly overexpressed in multiple solid tumors and is associated with poor prognosis [33, 34]. In this study, using database analyses we confirmed NQO1 overexpression in lung, breast, and colorectal cancer tissues, supporting its association with an unfavorable prognosis. Knocking out NQO1 significantly suppressed tumor growth both in vitro and in vivo. Additionally, we found that NQO1 is crucial for Cory‐mediated activation of PP2A and subsequent suppression of the PI3K/AKT and MAPK/ERK signaling pathways. These findings associate NQO1 overexpression with poor survival outcomes and support a model in which NQO1–PTPA association contributes to PP2A suppression and tumor‐promoting signaling, particularly in tumors with high NQO1 expression. This mechanism is distinct from previously reported NQO1‐mediated protein‐stabilizing functions, such as the stabilization of p53 or SIRT6, highlighting its unique role in carcinogenesis [17, 18].

**FIGURE 9:**
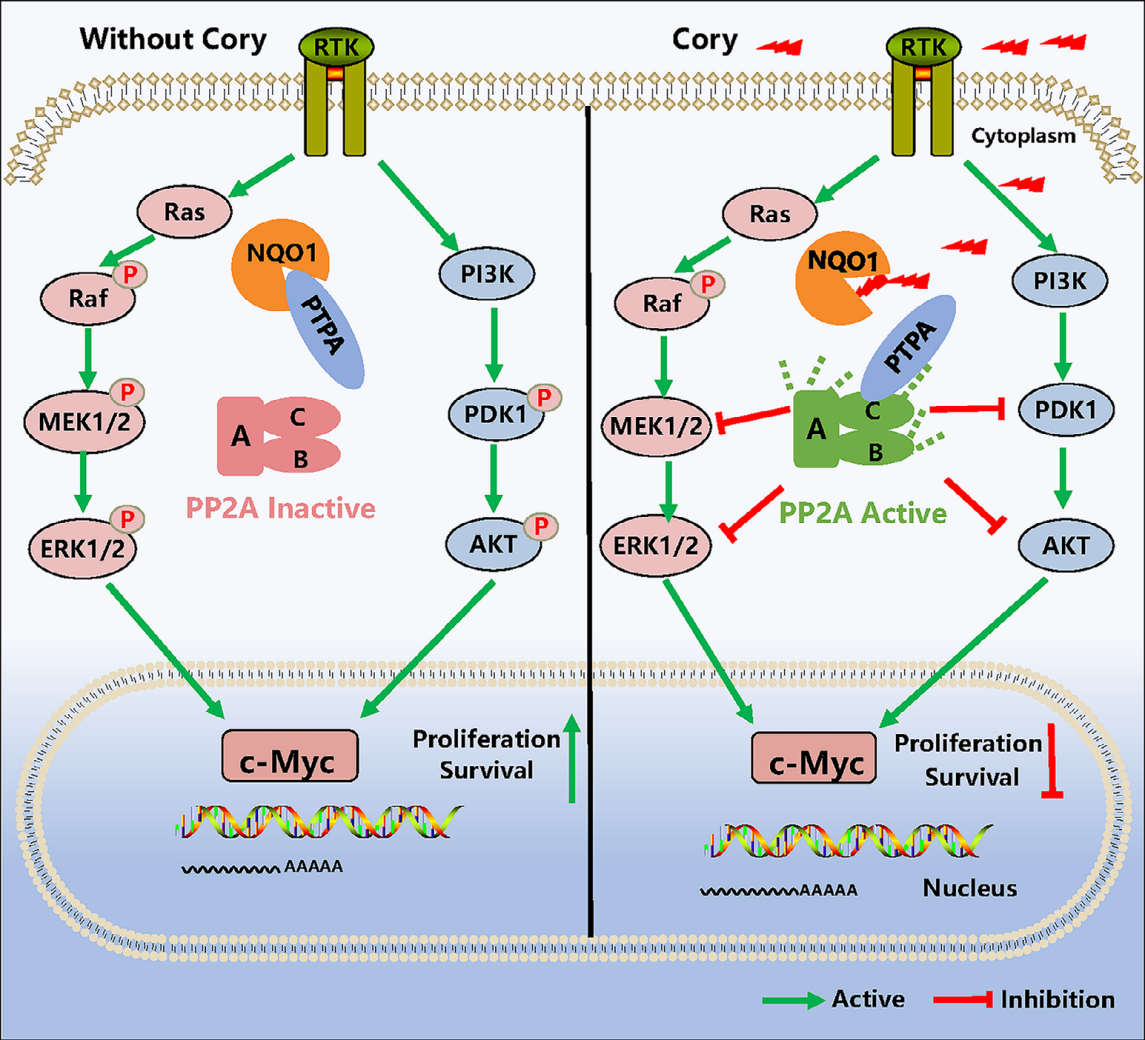
Schematic model illustrating the mechanism by which Cory inhibits tumor progression by targeting NQO1 and modulating the PTPA–NQO1/PP2A switch to activate PP2A. In the absence of Cory (left), NQO1 binds PTPA, keeping PP2A inactive and allowing sustained activation of the PI3K/AKT and Raf/MEK/ERK pathways, which promote tumor cell survival and proliferation. In the presence of Cory (right), Cory binds NQO1 and weakens the NQO1–PTPA association, facilitating PTPA association with PP2Ac and subsequent PP2A activation. Activated PP2A then dephosphorylates and inhibits key components of the PI3K/AKT and Raf/MEK/ERK pathways, leading to downregulation of the oncogenic driver c‐Myc and ultimately suppressing tumor cell survival and proliferation.

Our study elucidates the molecular mechanism underlying the interaction between NQO1 and PTPA, and further demonstrates how Cory disrupts this interaction to activate PP2A, thereby suppressing tumor growth. Specifically, amino acid residues 206‐215 and 241‐270 of PTPA mediate its interaction with NQO1. Previous studies have reported that residues 199‐218 of PTPA are essential for its interaction with the PP2Ac subunit [13]. These findings suggest that NQO1 occupies the 206‐215 region of PTPA, thereby limiting the ability of PTPA to associate with and activate PP2A. This interaction likely contributes to aberrant activation of the PI3K/AKT and MAPK/ERK signaling pathways, which are critical for cell survival and proliferation in NQO1‐overexpressing cell lines. Consistent with previous observations in liver cancer cells, NQO1 depletion enhanced PP2A activity [11]. Mechanistic studies revealed that Cory predominantly binds to the 1‐220 peptide segment of NQO1. Gly193 and His194 contribute to Cory recognition and are functionally critical for Cory‐mediated anticancer activity. Additionally, amino acid residues 191‐220 of NQO1 are critical for mediating its interaction with PTPA. Although the Cory–NQO1 interaction exhibits moderate micromolar affinity, our data are consistent with an NQO1‐dependent interface‐modulation model rather than classical high‐affinity competitive binding. The experimental data demonstrate that Cory weakens the cellular NQO1–PTPA association; we therefore propose that this effect may increase the availability of PTPA for PP2Ac engagement and subsequent PP2A activation. Whether local steric or conformational changes underlie this effect remains to be determined by structural studies. These findings provide critical mechanistic insights into tumor biology and offer promising avenues for therapeutic development (Figure 9).

The characterization of the PTPA–NQO1/PP2A regulatory axis carries several clinical implications. First, by demonstrating that NQO1 functions as a non‐enzymatic interaction partner to sequester PTPA, NQO1 emerges as a potential predictive biomarker for stratifying patients responsive to Cory. Second, because PP2A directly dephosphorylates AKT, targeting this axis circumvents resistance mechanisms driven by upstream PI3K or receptor tyrosine kinase mutations, which frequently complicate treatments in non‐small cell lung cancer. Finally, this mechanism provides a rationale for combination therapies. Combining Cory with NQO1‐bioactivatable agents such as β‐lapachone could yield synergistic antitumor effects, as Cory suppresses AKT‐mediated survival pathways while β‐lapachone exploits elevated NQO1 to induce oxidative stress and DNA damage [35].

Given the crucial contribution of phosphatases in maintaining cellular homeostasis, pharmacological activation of these enzymes has been demonstrated as a promising therapeutic approach when phosphatase genes are not genetically inactivated. Our study reveals a critical role of NQO1 in tumorigenesis, demonstrating that it promotes oncogenesis through suppression of PP2A activity, and identifies the NQO1–PTPA‐PP2A axis as a directly targetable pathway. The PTPA–NQO1/PP2Ac acts as a molecular switch that modulates PP2A activity via differential binding of PTPA to either NQO1 or PP2Ac. By targeting this newly identified switch, Cory emerges as a promising lead compound for the treatment of cancers with high NQO1 expression. Cory did not measurably inhibit NQO1 enzymatic activity or increase intracellular ROS under the tested conditions, supporting a mechanism primarily related to the non‐enzymatic protein‐interaction function of NQO1. Given that the PP2A inhibitor OA only partially reversed Cory‐induced tumor suppression, alternative PP2A‐independent pathways might coexist. Since NQO1 frequently acts as a protein‐stabilizing factor that interacts with and stabilizes downstream targets such as p53 [17], it will be highly valuable for future studies to investigate whether Cory systematically modulates these protein‐protein interactions to exert broader antitumor activities. This work raises several potential questions that warrant further investigation, including validation of the tumor‐suppressive effects in patient‐derived xenograft (PDX) models and clinical specimens. Although Cory‐induced changes in NQO1–PTPA association and downstream signaling were consistently observed in additional cancer cell lines, complete genetic loss‐and‐rescue experiments were mainly performed in A549 cells. Therefore, broader genetic validation in additional NQO1‐high tumor models remains an important future direction. While the current study focuses primarily on primary tumor growth, future studies should evaluate the anti‐metastatic activity of Cory in NQO1‐high metastatic models, with NQO1‐deficient MDA‐MB‐231 cells included as a mechanistically informative negative‐control model. Although Cory inhibited tumor growth in vivo at 10 mg/kg without obvious systemic toxicity, its effective concentrations in cellular assays remain relatively high. Moreover, direct LC‐MS/MS measurement of Cory concentrations in xenograft tumor tissues was not performed in this study, and future studies will be needed to better define the relationship between systemic pharmacokinetics, tumor exposure, and pharmacodynamic responses. Cory therefore represents a promising lead compound, and structural optimization or targeted delivery strategies may further enhance its therapeutic potential. Future structural studies of the NQO1–PTPA complex, together with tumor‐level pharmacokinetic analyses of Cory exposure, will be valuable for further defining the molecular basis and translational potential of this regulatory axis.

## 4 Conclusion

Our study demonstrates that NQO1 is markedly overexpressed in lung, colorectal, and breast cancers, highlighting its potential as a therapeutic target. We identify a novel oncogenic mechanism in which NQO1 binds to the PP2A‐activating protein PTPA, thereby suppressing the tumor‐suppressive activity of PP2A and promoting aberrant activation of the PI3K/AKT and MAPK/ERK signaling pathways in NQO1‐overexpressing cells. Our findings demonstrate that the distribution of PTPA between NQO1 and PP2Ac regulates PP2A activity. Mechanistically, we identify Cory as a direct NQO1‐targeting compound that binds NQO1 with moderate micromolar affinity, with Gly193 and His194 contributing to Cory recognition and functional regulation within the 1–220 peptide segment of NQO1. This interaction weakens the NQO1–PTPA association, which may facilitate PTPA engagement with PP2Ac and contribute to increased PP2A activity. Collectively, these findings provide mechanistic insights into tumor biology and establish Cory as a promising lead compound for the development of targeted anticancer therapeutics.

## 5 Materials and Methods

### 5.1 Chemicals and reagents

Corynoxine (purity ≥ 98.72%) was obtained from Chengdu Herbpurify Co., Ltd. (Chengdu, China). Okadaic acid was purchased from MedChemExpress (Monmouth Junction, NJ, USA; Catalog No. HY‐N6785). The primary antibodies used in this study were as follows: p‐PP2Ac (Abbkine, Cat# ABP55711), PP2Ac (CST, Cat# 2038), PP2Aa (CST, Cat# 2039), PP2Ab (CST, Cat# 4953), Caspase‐3 (CST, Cat# 9662), PTPA (Santa Cruz, Cat# 81607), NQO1 (Proteintech, Cat# 11451‐1‐AP), PDK1 (Proteintech, Cat# 18262‐1‐AP), p‐PDK1 (Proteintech, Cat# 29241‐1‐AP), p‐AKT (Proteintech, Cat# 29163‐1‐AP), AKT (Proteintech, Cat# 10176‐2‐AP), p‐MEK1/2 (CST, Cat# 2338), MEK1/2 (Proteintech, Cat# 11049‐1‐AP), p‐p44/42 MAPK (ERK1/2) (Thr202/Tyr204) (CST, Cat# 4370), ERK1/2 (Proteintech, Cat# 11257‐1‐AP), c‐Myc (Abbkine, Cat# ABP51011), GAPDH (Proteintech, Cat# 60004‐1‐Ig), Bax (Proteintech, Cat# 50599‐2‐Ig), Bcl2 (Proteintech, Cat# 68103‐1‐Ig), Cleaved PARP (Huabio, Cat# ET1608‐10), PARP (Huabio, Cat# ET1608‐56), Cleaved Caspase‐3 (Huabio, Cat# ET1602‐47), γ‐H2AX (Huabio, Cat# ET1602‐2) and Ki67 (Abbkine, Cat# ABP51674).

### 5.2 Cell Culture

A549, HT‐29, MCF‐7, BxPC‐3, SK‐OV‐3, and HEK 293T cell lines were obtained from the American Type Culture Collection (ATCC, USA). MCF‐7, BxPC‐3, SK‐OV‐3, and HEK 293T cells were cultured in Dulbecco's Modified Eagle Medium (DMEM, Gibco, USA) supplemented with penicillin (100 units/mL), streptomycin (100 µg/mL), and 10% fetal bovine serum (FBS, Gibco, USA). A549 cells were cultured in RPMI‐1640 medium (Gibco, USA), and HT‐29 cells were cultured in McCoy's 5A medium with the same supplements. All cells were maintained in a humidified atmosphere at 37°C with 5% CO_2_.

### 5.3 Cell Viability Assay and Colony Formation Assay

Cells were seeded at a density of 2 × 10^3^ cells/well in 100 µL of culture medium into 96‐well plates and allowed to adhere for 12 h. After treatment for the specified duration, 10 µL of CCK‐8 was added, and the absorbance was measured at 450 nm after a 4 h incubation period. Cells (300 cells/well) seeded in a 6‐well plate were treated with various Cory concentrations for a specified duration. After treatment, cells were cultured for 10–14 days, fixed in 4% paraformaldehyde for 20 min, stained with crystal violet (Solarbio, Beijing, China) for 20 min, and colony counting was conducted using ImageJ software (NIH, USA).

### 5.4 Flow Cytometry

The Apoptosis Detection Kit (Yeasen, Shanghai, China) was used to assess apoptosis. Briefly, cells were harvested and resuspended in 100 µL of binding buffer, followed by sequential addition of 5 µL Annexin V‐FITC and 10 µL PI staining solution. After a 15 min incubation, samples were supplemented with 300–500 µL of binding buffer and analyzed by flow cytometry within 1 h. Data were analyzed using FlowJo 10.4.1 software (BD Biosciences, USA). ROS was measured using a ROS Assay Kit (Beyotime, China). After detachment and collection, cells were resuspended in serum‐free culture medium containing 10 µM DCFH‐DA. The suspension was incubated at 37°C for 30 min protected from light, with gentle mixing every 15 min. Subsequently, cells were washed three times with serum‐free culture medium, resuspended in 300 µL PBS, and analyzed using a flow cytometer. Data were analyzed using FlowJo 10.4.1 software (BD Biosciences, USA).

### 5.5 Synthesis of Cory‐Biotin Probe

The Cory‐Biotin probe was synthesized by Wayen Biotech (Shanghai, China). Briefly, Corynoxine was first hydrolyzed with LiOH at room temperature to generate the corresponding carboxylic acid intermediate, which was subsequently coupled with biotin‐PEG4‐NH2 under EDCI/DMAP/Et3N‐mediated conditions in anhydrous DMF to afford the Cory‐Biotin probe. The final product was purified by silica gel column chromatography and characterized by MS and NMR. The complete synthetic route and detailed experimental procedure are provided in Method S1: Synthetic route and detailed synthesis of Cory‐Biotin probe.

### 5.6 Pull‐Down Assay and LC‒MS/MS Analysis

D‐Biotin (100 µM) or Cory‐Biotin (100 µM) was incubated with cell lysates at 4°C for 8 h. Streptavidin‐coated magnetic beads (#HY‐K0208, MedChemExpress, China) were added to capture the target proteins, followed by further incubation at 4°C for 4 h. Subsequently, the bound protein complexes were separated by SDS‐PAGE, silver‐stained, the entire gel lanes or specific differential bands were excised for LC–MS/MS analysis

### 5.7 Surface Plasmon Resonance (SPR) Assay

Recombinant His‐NQO1 and His‐NQO1‐DMut were diluted to 40 µg/mL using 10 mM sodium acetate buffer at pH 4.0. Subsequently, proteins were immobilized on CM5 sensor chips (#29‐1049‐88, Cytiva, Sweden) using an amine coupling reagent kit (#BR‐1000‐50, Cytiva, Sweden). A series of Cory concentrations were injected through the protein‐CM5 chip system. Binding interactions were detected and analyzed using the Biacore T200 platform (Cytiva, Sweden).

### 5.8 Cellular thermal shift assay (CETSA)

Cell lysates were subjected to five cycles of freeze‐thaw using liquid nitrogen and then divided into control and Cory‐treated samples (100 µM, 4 h). Samples were heated across a temperature range of 37–67°C for 3 min, followed by cooling to room temperature. Additionally, the samples were divided into several portions and treated with increasing concentrations of Cory for 4 h, followed by heating at 53°C for 3 min and cooling. For live‐cell CETSA, Cory‐treated cells (100 µM, 8 h) were heated (46–67°C) for 3 min and then cooled. Cell lysates were collected by performing five cycles of freeze‐thaw using liquid nitrogen. The resulting lysates were centrifuged at 12,000 × g for 20 min at 4°C and analyzed by immunoblotting after SDS‐PAGE.

### 5.9 Drug Affinity Responsive Target Stability (DARTS) Assay

DARTS was performed according to previously described protocols [36]. Cell lysates were adjusted to equal protein concentrations and incubated with increasing concentrations of Cory at room temperature for 1 h. A stock solution of pronase (10 mg/mL) was prepared, and pronase was added to the lysates at pronase‐to‐protein ratios of 0.1%, 0.2%, and 0.4% (w/w, pronase: total protein). Samples were then incubated for 20 min at 37°C and analyzed by SDS‐PAGE for immunoblotting.

### 5.10 Computational Docking and Modeling

Energy minimization of Cory was performed using Chem3D Ultra 12.0 software (PerkinElmer, USA). The structures of NQO1 (PDB: 1H69) and PTPA (PDB: 2IXM) were processed using DS 2019 software, by adding hydrogen atoms and completing missing residues. Molecular docking simulations of proteins and Cory were conducted using the LibDOCK module. Additionally, protein‐protein docking simulations between NQO1 and PTPA were performed using the CDOCKER algorithm.

### 5.11 Biolayer Interferometry (BLI) Assay

The binding affinity between NQO1 and PTPA was measured using BLI on an Octet K2 system (Sartorius, Germany). Briefly, biotinylated PTPA (generated by cleaving the GST tag from purified GST‐PTPA) was immobilized onto Super Streptavidin biosensors (Sartorius, Germany) and equilibrated in PBST buffer. For the association phase, the biosensors were immersed in a threefold serial dilution of purified His‐NQO1 (12.34, 37.03, 111.11, 333.33, and 1000 nM). This was followed by a dissociation phase in the PBST buffer. To eliminate non‐specific binding and buffer‐induced shifts, reference subtraction was performed using blank biosensors exposed to the same analyte concentrations. Binding kinetics and the *K*_D_ were analyzed using Octet Analysis Studio 13 software (Sartorius, Germany).

### 5.12 Real‑Time qRT‑PCR

Total RNA was extracted using the RNA Isolator Total RNA Extraction Reagent (Vazyme, R401‐01, China) according to the manufacturer's instructions. First‐strand cDNA was synthesized with oligo(dT) primers using HiScript II Q RT SuperMix for qPCR (+gDNA wiper) (Vazyme, R223‐01, China). Quantitative PCR (qPCR) was performed using ChamQ SYBR qPCR Master Mix (Vazyme, Q311‐02, China), with GAPDH serving as the reference gene for normalization. The primer sequences used for qPCR were as follows:

*NQO1*:

forward 5′‐AAGCCGCAGACCTTGTGATATTCC‐3′,
reverse 5′‐CATGGCAGCGTAAGTGTAAGCAAAC‐3′.

*PTPA*:

forward 5′‐ACTCCAACCAGCTGTGGAAC‐3′,
reverse 5′‐AGTGCTGGATCACAGGGAA‐3′.

### 5.13 siRNA Transfection

The siNC and siNQO1 (sc‐37139) were purchased from Santa Cruz Biotechnology. A549 cells were seeded into 6‐well plates and transfected with siNC or siNQO1 using Lipofectamine 2000 reagent (Invitrogen, Carlsbad, CA, USA) according to the manufacturer's instructions. After transfection, the cells were cultured for 24 h and used for subsequent experiments.

### 5.14 CRISPR/Cas9 Knockout Cell Lines

The NQO1‐knockout (KO) cell lines were generated using CRISPR/Cas9 genome editing as previously described [37]. Cells were transfected with the pSpCas9(BB)‐2A‐Puro (PX459) plasmid to achieve the deletion of NQO1 in A549 cells. The pX459 plasmid encodes the Cas9 protein and specific small guide RNA (sgRNA) sequences targeting NQO1. The synthetic oligonucleotides were cloned into the linearized pX459 vector using BbsI restriction endonuclease (NEB#R0539V) to generate the plasmid. Designed sgRNA sequences were synthesized based on sequences generated using the sgRNA design tool (https://zlab.bio/guide‐design‐resources) as listed in Table S3. At 36 h post‐transfection, cells were selected with a 1.0 µg/mL dose of puromycin (ST551, Beyotime, China) for 3–5 days. Single clones were isolated, expanded, and verified by Sanger sequencing and Western blot analysis.

### 5.15 Lentiviral Construct and Rescue Expression

NQO1^WT^ or NQO1^DMut^ (the double mutant of NQO1, G193A/H194A) sequences were synthesized and cloned into the pLV‐NQO1^WT^ or pLV‐NQO1^DMut^ expression vectors, respectively. These plasmids were co‐transfected with psPAX2 and pMD2.G into HEK 293T cells. Supernatants containing lentiviral particles were harvested to determine virus titers and then utilized to infect NQO1 knockout (KO) A549 cells. Flow cytometry was employed to selectively isolate EGFP‐positive populations. Real‐time RT‐PCR was conducted to assess NQO1 expression in the cells, and protein immunoblotting was employed to analyze NQO1 protein expression.

### 5.16 Bioinformatic Analyses

The GSE30219 dataset (based on the GPL570 platform) was downloaded from the Gene Expression Omnibus (GEO) database. ‘limma’ R package was used to identify differentially expressed genes (DEGs) [38], and all DEGs with an FDR lower than 0.05 and a |log_2_ FC| of more than 1 were retained for further analysis. The results are summarized in Table S2. NQO1 expression in cancerous and adjacent normal tissues was also analyzed using the Human Protein Atlas (HPA, http://www.proteinatlas.org) database. GEPIA (http://gepia2.cancer‐pku.cn) is a web‐based tool used to analyze TCGA clinical data and GTEx data to compare NQO1 expression between human cancers and normal tissue. The prognostic significance of NQO1 expression in various cancers was evaluated using the Kaplan–Meier Plotter database (http://kmplot.com/analysis/).

### 5.17 PP2A and NQO1 Activity Assay

The PP2A phosphatase activity assay was performed as previously described [39]. Cell lysates (100 µg) were mixed with 40 µL Protein A Sepharose bead (Santa Cruz Biotechnology, sc‐2001) and 2 µL anti‐PP2A C‐subunit antibody (Sigma–Aldrich, 05‐421) in a 500 µL volume with pNPP Ser/Thr Detection Buffer (Sigma–Aldrich, 20‐179). The mixture was incubated at 4°C for 2 h. The beads were then washed three times with TBS, followed by one wash with pNPP Ser/Thr Detection Buffer. Next, phosphorylated serine phosphopeptide (Sigma–Aldrich, 12‐219) was added to the beads and pNPP Ser/Thr assay buffer, reaching a final concentration of 500 µM. The mixture was incubated at 30°C for 10 min. Then, 25 µL of the reaction supernatant was mixed with 100 µL Malachite Green Phosphate Detection Solution (Sigma–Aldrich, 20‐105 and 20‐104) in a 96‐well plate. The mixture was incubated at room temperature for 10 min, and absorbance was measured at 650 nm using a Varioskan plate reader (Thermo Fisher Scientific, USA) and compared with a phosphate standard (Sigma–Aldrich, 20‐103). The NQO1 enzymatic activity assay was performed as previously described [40]. For the cell lysate‐based assay, 50 µg of total cell lysate was used for each reaction. For the recombinant protein‐based assay, purified His‐NQO1 protein was pre‐incubated with Cory at the indicated concentrations for 30 min at room temperature. Briefly, the reaction mixture contained cell lysates (50 µg) or purified His‐NQO1 protein (50 ng), FAD (5 µM), NADH (200 µM), and Tris‐HCl buffer (25 mM Tris‐HCl, pH 7.5, 0.7 mg/mL BSA, 0.01% Tween 20) in a total volume of 200 µL. Equal concentrations of DMSO were included in all groups. NQO1 enzymatic activity was measured by monitoring the decrease in NADH absorbance at 340 nm using a microplate reader. Relative NQO1 activity was calculated from the linear rate of NADH consumption and normalized to the corresponding vehicle‐treated control.

### 5.18 Protein Expression, Purification, and Pull‐Down

For the purification of His‐tagged proteins, the constructed pET28a (+) plasmid vectors encoding His‐NQO1, His‐NQO1‐G193A, His‐NQO1‐H194A, His‐NQO1‐DMut (the double mutant of NQO1, G193A/H194A), and His‐NQO1‐Δ191‐220 constructs were individually transformed into Escherichia coli BL21(DE3) competent cells (Solarbio, Beijing, China) for the production of recombinant proteins. Transformed cells were cultured until reaching an OD600 of 0.7, induced with 0.5 mM IPTG, and cultured at 16°C for 24 h. Following induction, cells were harvested by centrifugation at 4000 rpm for 20 min at 4°C. The cell pellets were resuspended in lysis buffer (20 mM HEPES, 250 mM NaCl, 10 mM imidazole) at a ratio of 6 mL/g of wet cell mass and lysed by sonication. Subsequently, Ni‐NTA resin (GE Healthcare, Chicago, IL, USA) was used to bind the proteins, and a gradient elution with imidazole wash buffer was performed. The proteins eluted with 400 mM imidazole were collected for downstream applications. In a His pull‐down assay, Sepharose beads were thoroughly washed with affinity buffer (50 mM MOPS, 10 mM Na2HPO4, 50 mM NaCl). Subsequently, His‐NQO1 (250 µg) was introduced, and in a molar ratio of 1:1.2 with GST, GST‐PTPA, or their respective mutants were added. The reaction mixture was supplemented with the affinity buffer to reach a total volume of 2 mL and then incubated at 4°C for 8 h. After centrifugation (4°C, 3000 rpm, 5 min), the supernatant was carefully removed, and SDS loading buffer was incorporated. The samples were boiled for 10 min, followed by identification using Coomassie Brilliant Blue staining. For the purification of GST‐tagged proteins, pGEX‐4T3 plasmid vectors encoding GST‐PTPA, GST‐PTPA‐△206‐215, and GST‐PTPA‐△241‐270 were constructed. Each plasmid, including the pGEX‐4T3 vector (for GST purification), was individually transformed into the BL21(DE3) bacterial strain. Transformed cells were cultured until reaching an OD600 of 0.7, induced with 0.5 mM IPTG, and cultured at 16°C for 24 h. Post‐induction, cells were collected by centrifugation at 4000 rpm for 20 min at 4°C. Cell pellets were resuspended in lysis buffer composed of 20 mM Tris‐HCl (pH 7.0), 50 mM NaCl, 0.5 mM EDTA, 1 mM dithiothreitol (DTT), 1 mM protease inhibitor cocktail, and 1 mM PMSF. Lysates were cleared by centrifugation at 9500 rpm for 30 min, and the supernatant was mixed with glutathione‐agarose beads (Beijing ComWin Biotech). After rotating at 4°C for 3 h, bound proteins were eluted and collected for analysis. GST pull‐down assay was performed as previously described [41].

### 5.19 Immunofluorescence Analysis

Cells were fixed with 4% paraformaldehyde (P1110, Solarbio, China) for 15 min, followed by permeabilization with 0.5% Triton X‐100 (P0096, Beyotime, China) for 30 min. Subsequently, cells were blocked with 5% BSA at room temperature for 30 min, and incubated overnight with the primary antibody at 4°C. Cells were then incubated with the appropriate fluorescent secondary antibody for 2 h at room temperature. The cells were further counterstained with DAPI (C1005, Beyotime, China) for 10 min. Images were acquired using a confocal laser scanning microscope.

### 5.20 Western Blot and Co‐Immunoprecipitation (co‐IP) Analysis

Protein expression levels or modifications in tissues or cells were assessed through Western blot analysis, employing methodologies as described in our previous protocols [42]. After treatment, cell lysates were incubated with bait antibodies overnight at 4°C. Subsequently, protein A/G agarose beads (P2012, Beyotime, China) were added and incubated at 4°C for 3 h. The resulting complexes were washed five times with PBS, followed by immunoblot analysis using the specified antibodies.

### 5.21 Xenograft Tumor Growth In Vivo

BALB/c nude mice (6–8 weeks old) were obtained from Beijing Weitong Lihua Experimental Animal Technology Co., Ltd. A549, A549 NQO1 KO, A549 NQO1^WT^, and A549 NQO1^DMut^ cells were suspended at a concentration of 5 × 10^6^ cells in a 50% Matrigel mixture and subcutaneously injected into the dorsal flank of the mice. When tumors reached a tumor volume of approximately 50 mm^3^, mice were randomly assigned to different treatment groups. Unless otherwise stated, treatments were administered via tail vein injection every two days for a total of six doses. Tumor growth was monitored using calipers, and tumor volume was calculated using the formula: V = 0.52 × length × width^2^. Body weights were recorded regularly throughout the study. At the end of the study, mice were euthanized, and tumors were excised and weighed. Additionally, tissues from the heart, lung, liver, kidney, and spleen were collected. Before randomization, mice lacking tumors or bearing tumors exceeding twice the mean volume were excluded. Staining with hematoxylin and eosin (H&E) was used for histological examination. TUNEL staining and immunohistochemistry (IHC) analysis were performed as previously reported [43]. All animal procedures were performed in accordance with the guidelines approved by the Institutional Animal Care and Use Committee of Taizhou University (TZXY‐2023‐20231074).

### 5.22 Pharmacokinetic Analysis

For exploratory pharmacokinetic (PK) evaluation, BALB/c nude mice (6–8 weeks; Hunan SJA Laboratory Animal Co., Ltd.; Animal ethics approval: Servicebio Animal Welfare No. 2026096) were randomly assigned to 9 groups (n = 3/group), using a minimal sample size in accordance with the 3Rs (Reduction) animal welfare principle. Following baseline serum collection (0 min), mice received a single intravenous injection of Cory (10 mg/kg). Destructive blood sampling was performed at 5, 15, and 30 min, followed by 1, 2, 4, 8, and 24 h post‐injection to obtain serum. Serum Cory concentrations were quantified via LC‐MS/MS (UltiMate 3000 RS and TSQ Quantum, Thermo Fisher Scientific, USA) using Xcalibur 3.0 software. Standard curves were fitted by linear regression (1/x^2^ weighting). Key PK parameters, including *C_max_, T_max_, t_1/2_, AUC_0‐t_*, and *AUC_0‐∞_*, were directly derived from the concentration and time profiles using non‐compartmental analysis.

### 5.23 Statistical Analysis

Statistical analyses were performed using GraphPad Prism 7.0 software. Data are presented as mean ± standard deviation (SD). All experiments were independently repeated at least three times unless otherwise stated. For comparisons between two groups, an unpaired two‐tailed Student's t‐test was used. For multiple‐group comparisons, one‐way ANOVA followed by Dunnett's or Tukey's post hoc test was applied as appropriate. Two‐way ANOVA was used for analyses involving two independent variables, including time‐course and dose–response experiments. Survival curves were analyzed using the log‐rank test. *P*‐values less than 0.05 were considered statistically significant.

## Author Contributions

**Guoqing Hou**: Investigation, formal analysis, methodology, data curation, Writing – original draft; **Li Lin**: Investigation, formal analysis, methodology, data curation; **Mengjie Xia** and **Ruohan Liang**: Writing – reviewing and editing; **Yueyue Zhang**: Software, data curation; **Linna Du** and **Xuan Cao**: Conceptualization, writing – review and editing, supervision, funding acquisition. All authors read and approved the final paper.

## Conflicts of Interest

The authors declares no conflicts of interest.

## Supporting information




**Supporting File 1**: advs77362‐sup‐0001‐SuppMat.docx.

**Supporting File 2**: advs77362‐sup‐0002‐TableS1.xlsx.

**Supporting File 3**: advs77362‐sup‐0003‐TableS2.xlsx.

**Supporting File 4**: advs77362‐sup‐0004‐TableS3.xlsx.

## Data Availability

The data that support the findings of this study are available from the corresponding author upon reasonable request.
