# Supplementary material for: Corynoxine Inhibits Tumor Growth via Targeting NQO1 and Modulating the PTPA–NQO1/PP2A Switch to Activate PP2A
Source: Adv Sci (Weinh). 2026 Aug 20;13(60):e77362. doi: 10.1002/advs.77362 (PMC13491527; doi:10.1002/advs.77362)
Supplement: Supplementary file 1 [file ADVS-13-e77362-s001.docx]

**Corynoxine inhibits tumor growth via targeting NQO1 and modulating the PTPA–NQO1/PP2A switch to activate PP2A**

**Guoqing Hou^1,2#^, Li Lin^1#^, Mengjie Xia^1^, Ruohan Liang****^1^, Yueyue Zhang^1^, Linna Du^1*^, Xuan Cao^1^****^,3*^**

1. School of Medicine, Taizhou University, Taizhou, 318000, Zhejiang, China.

2. The Affiliated Cancer Hospital of Zhengzhou University & Henan Cancer Hospital, Zhengzhou,450008, Henan, China.

3. Department of Basic Medicine and Medical Laboratory Science, School of Medicine, Zhejiang Key Laboratory of New Drug Development for Central Nervous System Diseases, Taizhou University, Taizhou, 318000, Zhejiang, China.

**^#^** These authors contributed equally to this work.

**^*^**Corresponding author:

Xuan Cao, Email: [caoxuan@tzc.edu.cn](mailto:caoxuan@tzc.edu.cn); Phone: +86-150-6866-9696.

Linna Du, Email: [dulina1028@126.com](mailto:dulina1028@126.com); Phone: +86-136-4381-9697.

**Supplementary materials**

**Figure S1.** Cory exhibits anticancer activity *in vitro*. (A-B) Colony formation assay in BxPC-3 and SK-OV-3 cells treated with Cory (0–200 μM) for 24 h. Representative images (A) and quantitative analysis of relative colony numbers (B) are shown. (C-D) Representative immunoblots (C) and corresponding densitometric quantification (D) of Caspase-3, Cleaved Caspase-3, PARP, Cleaved PARP, and γ-H2AX expression in A549, HT-29, and MCF-7 cells following 24 h of Cory treatment (0–400 μM). GAPDH was used as the loading control. (N.D., not detected; MCF-7 cells lack endogenous Caspase-3 due to *CASP3* deletion). (E) Quantitative immunoblot analysis of Bax and Bcl2 in A549, HT-29, and MCF-7 cells treated with Cory (0–400 μM) for 24 h. (F) Western blot analysis of PP2Ac, PDK1, AKT, MEK1/2, and ERK1/2 in HT-29 and MCF-7 cells treated with 100 μM Cory and OA (0–10 nM) for 24 h. (G) Quantitative immunoblot analysis of PP2Ac, PDK1, AKT, MEK1/2, and ERK1/2 (normalized to GAPDH) in A549, HT-29, and MCF-7 cells co-treated with 100 μM Cory and OA (0–10 nM) for 24 h. (H-I) Representative immunoblots (H) and corresponding densitometric quantification (I) of PP2Aa and PP2Ab expression in A549, HT-29, and MCF-7 cells following 24 h of Cory treatment (100 μM). GAPDH was used as the internal loading control. Data are presented as mean ± SD from at least three independent biological replicates. Representative images are shown from at least three independent experiments. Statistical significance was determined using unpaired two-tailed Student’s t-test or one-way ANOVA followed by Dunnett’s or Tukey’s post hoc test as appropriate; **P* < 0.05; ns, not significant.

**Figure S2.** Cory suppresses tumor growth *in vivo*. (A) H&E staining of heart, liver, spleen, lung, and kidney tissues from mice treated with Cory (0, 5, or 10 mg/kg) or Cory (10 mg/kg) combined with okadaic acid (OA, 2 μg/kg). Representative images are shown from tumor-bearing mice in each treatment group. (B) Quantification of TUNEL fluorescence intensity in tumor tissues from the indicated treatment groups. Fluorescence intensity was quantified using ImageJ and normalized to the control group. (C) Quantitative analysis of IHC staining intensity for Ki67, p-PP2Ac, p-AKT, and p-MEK1/2 in tumor tissues from the indicated treatment groups. Staining intensity was quantified and normalized to the control group. (D–E) Western blot analysis of p-PP2Ac, PP2Ac, p-PDK1, PDK1, p-AKT, AKT, p-ERK1/2, ERK1/2, p-MEK1/2, and MEK1/2 in tumor tissues from the indicated treatment groups (D), with quantitative analysis of phosphorylated-to-total protein ratios shown in (E). GAPDH was used as the loading control. For panels A–E, tumor tissues from three mice per group were analyzed. Scale bar: 100 μm; magnification: ×20. Data are presented as mean ± SD. Statistical significance was determined using one-way ANOVA followed by Dunnett’s or Tukey’s post hoc test as appropriate; **P* < 0.05; ns, not significant.

**Figure S3**. Structural Characterization and Purity Analysis of Cory-Biotin. (A) Mass spectrometry of Cory-Biotin. The observed m/z 799.4436 [M+H]⁺ corresponds to the theoretical molecular mass of Cory-Biotin (799.4423 Da) with an error of 1.62 ppm. (B) ¹H NMR spectrum of Cory-Biotin. The purity of the compound is estimated to be >95% based on spectral resolution and absence of significant impurity peaks.

**Figure S4**. Validation of Cory interaction with NQO1. (A) CETSA of A549 cell-derived proteins incubated with Cory (0–400 μM) at 4 °C for 4 h, followed by heating at 53 °C. Relative NQO1 levels are shown. (B) Predicted binding mode between Cory and NQO1 from computational docking. (C) Hydrogen-bond acceptor and donor map of NQO1 upon Cory binding. (D-G) Coomassie Blue-stained SDS-PAGE of purified wild-type and mutant NQO1 proteins. (H–I) Effects of NQO1 mutations on Cory binding. (H) Densitometric analysis of streptavidin pull-down assays of HA-tagged NQO1 variants (WT, G193A, H194A, and G193A/H194A) expressed in HEK 293T cells and incubated with Cory-Biotin, normalized to WT. (I) Quantification of streptavidin pull-down assays of purified His-tagged NQO1 variants incubated with Cory-Biotin, normalized to WT. (J) NQO1 activity was determined in lysates prepared from A549 cells following Cory (100 μM) and normalized to the vehicle-treated control. (K) Purified recombinant NQO1 was incubated with Cory at the indicated concentrations (0–400 μM), and NQO1 activity was measured in a cell-free system. Relative activity was normalized to the vehicle-treated control. (L) ROS levels in A549 cells treated with 100 μM Cory, measured by flow cytometry. Data are presented as mean ± SD from three independent biological replicates. Representative images are shown from at least three independent experiments. Statistical significance between two independent groups was determined using an unpaired two-tailed Student’s t-test. Comparisons among multiple groups were analyzed using one-way ANOVA followed by Dunnett's post hoc test; **P* < 0.05; ns, not significant.

**Figure S5**. Cory activates PP2A by attenuating the NQO1–PTPA interaction. (A-C) A549, HT-29, and MCF-7 cells were treated with Cory (0–200 μM) for 24 h. Western blot (A) and quantitative analysis (B, normalized to GAPDH) of NQO1 and PTPA protein levels; mRNA levels of NQO1 and PTPA normalized to GAPDH (C). (D-E) Co-IP analysis of the endogenous interaction between NQO1 and PTPA in HT-29 and MCF-7 cells treated with Cory (0 or 100 μM) for 24 h (D), with quantification of NQO1 co-immunoprecipitated with PTPA and PTPA co-immunoprecipitated with NQO1 in A549, HT-29, and MCF-7 cells (E). (F) Quantification of Co-IP assays showing the interaction between HA-NQO1 and FLAG-tagged PTPA variants, including WT, Δ206–215, and Δ241–270. (G) Quantification of Co-IP assays showing the interaction between FLAG-PTPA and HA-tagged NQO1 variants, including WT and Δ191–220. (H-L) Coomassie Blue-stained SDS-PAGE of purified GST, GST-PTPA, GST-PTPA-Δ241–270, GST-PTPA-Δ206–215, and NQO1-Δ191–220 proteins. (M) Quantification of His pull-down assays showing the relative binding of GST-PTPA variants, including FL, Δ206–215, and Δ241–270, to His-NQO1. (N) Quantification of GST pull-down assays showing the relative binding of His-NQO1 FL or His-NQO1-Δ191–220 to GST-PTPA. (O) PTPA was immunoprecipitated and PP2Ac was detected by immunoblotting. Relative PP2Ac co-immunoprecipitated with PTPA was significantly increased in siNQO1 cells compared with siNC cells. (P) Quantification of Co-IP assays showing the interaction between PTPA and PP2Ac in A549 cells transfected with siNC or siNQO1 and treated with Cory (0 or 100 μM) for 24 h. (Q) PP2A activity in A549 cells transfected with siNC or siNQO1 and treated with Cory (0 or 100 μM) for 24 h. (R) Quantitative immunoblot analysis of NQO1, PP2Ac, PDK1, AKT, MEK1/2, ERK1/2, and c-Myc in A549 cells transfected with siNC or siNQO1 and treated with Cory (0 or 100 μM) for 24 h (normalized to GAPDH). Data are presented as mean ± SD from three independent biological replicates. Representative images are shown from at least three independent experiments. Statistical significance between two independent groups was determined using an unpaired two-tailed Student’s t-test. Comparisons among multiple groups were analyzed using one-way ANOVA followed by Dunnett's post hoc test; **P* < 0.05; ns, not significant.

**Figure S6.** High expression of NQO1 contributes to tumorigenesis. (A) Representative immunohistochemical staining images showing NQO1 protein expression in adjacent non-tumor tissues and tumor tissues from human breast and colorectal tissue microarrays. Images were obtained from the Human Protein Atlas; lower panels show magnified views of the boxed regions.

(B) Unpaired analysis of *NQO1* mRNA expression in breast cancer and colorectal cancer tissues compared with normal tissues from the TCGA/GTEx dataset. NQO1 expression was significantly increased in breast cancer (BRCA; tumor, n = 1085; normal, n = 291) and colorectal cancer/colon adenocarcinoma (COAD; tumor, n = 275; normal, n = 349). (C) Paired analysis of *NQO1* mRNA expression in tumor tissues and matched adjacent non-tumor tissues from breast cancer and colorectal cancer patients in the TCGA dataset. NQO1 expression was higher in paired tumor tissues than in adjacent normal tissues in both breast cancer (n = 113 pairs) and colorectal cancer (n = 50 pairs). (D) Kaplan–Meier overall survival analyses of patients with breast cancer or colorectal cancer stratified according to NQO1 expression level using the Kaplan–Meier Plotter database. (E) Quantitative Western blot analysis of NQO1, PP2Ac, PDK1, AKT, MEK1/2, ERK1/2, and c-Myc in WT and NQO1 KO A549 cells. (F) ROS levels in WT and NQO1 KO A549 cells measured by flow cytometry. (G) Quantification of PP2Ac co-immunoprecipitated with PTPA in WT and NQO1 KO A549 cells. (H) Quantification of IHC staining intensity and downstream signaling proteins (Ki67, p-PP2Ac, p-AKT, p-MEK1/2, and c-Myc) in xenograft tumors derived from WT and NQO1 KO A549 cells. (I-J) Western blot (I) and quantitative analysis (J, normalized to GAPDH) of PP2Ac, PDK1, AKT, ERK1/2, MEK1/2, and c-Myc in tumor tissues. Data are presented as mean ± SD from three independent biological replicates for *in vitro* experiments or from six mice per group for *in vivo* experiments. Representative images are shown from at least three independent experiments. Statistical significance between two independent groups was determined using an unpaired two-tailed Student’s t-test, while paired clinical samples were analyzed using a paired t-test. Survival analysis was evaluated using the log-rank test; **P* < 0.05; ns, not significant.

**Figure S7.** Cory-mediated tumor suppression depends on NQO1. (A) Quantitative immunoblot analysis of NQO1, PP2Ac, PDK1, AKT, MEK1/2, ERK1/2, and c-Myc in A549 NQO1 KO cells treated with Cory (0 or 100 μM) for 24 h (normalized to GAPDH). (B) Quantitative immunoblot analysis of Bax and Bcl2 in WT and NQO1 KO A549 cells treated with Cory (0 or 100 μM) for 24 h (normalized to GAPDH). (C) Co-IP analysis and quantification of the interaction between PTPA and PP2Ac in WT and NQO1 KO A549 cells treated with Cory (0 or 100 μM). (D) TUNEL assay showing apoptotic cell death in xenograft tumor tissues from control and Cory-treated (10 mg/kg) groups. Quantification is shown as relative TUNEL fluorescence intensity. (E) IHC analysis and quantification of Ki67, p-PP2Ac, p-AKT, p-MEK1/2, and c-Myc in xenograft tumor sections from control and Cory-treated (10 mg/kg) groups. (F-G) Western blot (F) and quantitative analysis (G, normalized to GAPDH) of PP2Ac, PDK1, AKT, MEK1/2, ERK1/2, and c-Myc in tumor tissues from A549 NQO1 KO xenograft mice. Data are presented as mean ± SD from three independent biological replicates for *in vitro* experiments or from six mice per group for *in vivo* experiments. Representative images are shown from at least three independent experiments. Statistical significance between two independent groups was determined using an unpaired two-tailed Student’s t-test. Comparisons involving two independent variables were analyzed using two-way ANOVA; **P* < 0.05; ns, not significant.

**Figure S8.** Gly193 and His194 of NQO1 are required for anticancer activity of Cory. (A) Quantitative analysis of NQO1 pulled down by Cory-Biotin in A549 cells expressing NQO1^WT^ and NQO1^DMut^. (B) Quantitative immunoblot analysis of Bax and Bcl2 in NQO1^WT^ and NQO1^DMut^ A549 cells treated with Cory (0 or 100 μM) for 24 h (normalized to GAPDH). (C) Quantitative immunoblot analysis of NQO1, PP2Ac, PDK1, AKT, MEK1/2, ERK1/2, and c-Myc in NQO1^WT^ and NQO1^DMut^ A549 cells treated with Cory (0 or 100 μM) for 24 h (normalized to GAPDH). (D) Co-IP analysis and quantification of PP2Ac–PTPA and NQO1–PTPA interactions in A549 cells expressing NQO1^WT^ and NQO1^DMut^ treated with Cory (0 or 100 μM). (E) Quantification of TUNEL-positive apoptotic cells in xenograft tumor tissues derived from A549 cells expressing NQO1^WT^ and NQO1^DMut^ after vehicle or Cory treatment (10 mg/kg). (F) IHC analysis and quantification of Ki67, p-PP2Ac, p-AKT, p-MEK1/2, and c-Myc in tumor sections derived from NQO1^WT^ and NQO1^DMut^ A549 xenograft mice treated with Cory (10 mg/kg) or vehicle control. Staining intensity was quantified and normalized to the corresponding control group. (G-H) Western blot (G) and quantitative analysis (H, normalized to GAPDH) of PP2Ac, PDK1, AKT, MEK1/2, ERK1/2, and c-Myc in tumor tissues from NQO1^WT^ and NQO1^DMut^ A549 xenograft mice. Data are presented as mean ± SD from three independent biological replicates for *in vitro* experiments or from six mice per group for *in vivo* experiments. Representative images are shown from at least three independent experiments. Statistical significance between two independent groups was determined using an unpaired two-tailed Student’s t-test. Comparisons involving two independent variables were analyzed using two-way ANOVA; **P* < 0.05; ns, not significant.

**Supporting** **Method S1: Synthetic route and detailed synthesis of Cory-Biotin probe.**

The Cory-Biotin probe was synthesized by Wayen Biotech (Shanghai, China) through a two-step route. Corynoxine (1) was first hydrolyzed to afford the demethylated intermediate (2), which was subsequently coupled with biotin-PEG₄-NH₂ to yield the Cory-Biotin probe (3).

**Scheme S1. Synthetic route of the Cory-Biotin probe.**

**Reagents and conditions:** (a) LiOH, THF/H₂O, room temperature, 12 h; (b) biotin-PEG₄-NH₂, EDCI, DMAP, Et₃N, anhydrous DMF, room temperature, 48 h.

**Detailed synthesis of the Cory-Biotin probe**

**Step 1. Synthesis of demethylated intermediate 2**

Corynoxine (100 mg, 0.26 mmol) was dissolved in tetrahydrofuran (THF, 5 mL). A solution of lithium hydroxide (LiOH, 125 mg, 20 equiv.) in water (5 mL) was then added to the reaction mixture. The mixture was stirred at room temperature for 12 h. After complete consumption of the starting material, as monitored by thin-layer chromatography (TLC), the organic solvent was removed under reduced pressure. The residue was diluted with water (10 mL) and extracted with ethyl acetate (5 mL × 3). The combined organic layers were washed with brine, dried over anhydrous sodium sulfate, and concentrated in vacuo to afford demethylated intermediate 2 as a white solid (dichloromethane/methanol = 20:1, Rf = 0.25). The crude intermediate was used directly in the next step without further purification.

**Step 2. Synthesis of Cory-Biotin probe 3**

Crude intermediate 2 (50 mg, 0.135 mmol) was dissolved in anhydrous DMF (3 mL). EDCI (39 mg, 0.2 mmol), DMAP (8 mg, 0.067 mmol), and biotin-PEG₄-NH₂ (75 mg, 0.168 mmol) were added to the solution, followed by triethylamine (Et₃N, 56 μL, 0.4 mmol). The reaction mixture was stirred at room temperature for 48 h. After completion of the reaction, as monitored by TLC, the solvent was removed under reduced pressure. The residue was partitioned between water (5 mL) and dichloromethane (DCM), and the aqueous phase was extracted with DCM (3 mL × 3). The combined organic extracts were washed with brine, dried over anhydrous sodium sulfate, and concentrated. The crude product was purified by silica gel column chromatography using a stepwise gradient of dichloromethane/methanol from 20:1 to 3:1 (v/v), affording Cory-Biotin probe 3 as a white solid (35 mg, 32% yield).

**Supporting Information Table 1:** Cory-Biotin and D-Biotin pull-down proteomic summary

**Supporting Information Table 2**: GSE30219 differentially expressed genes

**Supporting Information Table 3**: Cory-Biotin differential-band proteomic summary
